# Mutation in IR or IGF1R produces features of long-lived mice while maintaining metabolic health

**DOI:** 10.1172/jci.insight.189683

**Published:** 2025-11-11

**Authors:** Ulalume Hernández-Arciga, Jun Kyoung Kim, Jacob L. Fisher, Alexander Tyshkovskiy, Alibek Moldakozhayev, Catherine Hall, Souvik Ghosh, Yashvandhini Govindaraj, Ian J. Sipula, Jake Kastroll, Diana Cooke, Jinping Luo, Jonathan K. Alder, Stacey J. Sukoff Rizzo, Gene P. Ables, Eunhee Choi, Vadim N. Gladyshev, Michael J. Jurczak, Marc Tatar, Andrey A. Parkhitko

**Affiliations:** 1 Aging Institute of UPMC and University of Pittsburgh, Pittsburgh, Pennsylvania, USA.; 2 Dietrich School of Arts & Sciences, University of Pittsburgh, Pennsylvania, USA.; 3 Division of Genetics, Department of Medicine, Brigham and Women’s Hospital, Harvard Medical School, Boston, Massachusetts, USA.; 4 Belozersky Institute of Physico-Chemical Biology, Moscow State University, Moscow, Russia.; 5 Department of Neurology and Neurosurgery, McGill University, Montreal, Quebec, Canada.; 6 Metabolic Disorders and Complications Program, and Brain Repair and Integrative Neuroscience Program, Research Institute of the McGill University Health Centre, Montreal, Quebec, Canada.; 7 Broad Institute of MIT and Harvard, Cambridge, Massachusetts, USA.; 8 Department of Pathology and Cell Biology, Vagelos College of Physicians and Surgeons, Columbia University, New York, New York, USA.; 9 Department of Human Genetics, School of Public Health,; 10 Center for Metabolism and Mitochondrial Medicine, Department of Medicine, and; 11 Division of Endocrinology and Metabolism, Department of Medicine, University of Pittsburgh, Pittsburgh, Pennsylvania, USA.; 12 Orentreich Foundation for the Advancement of Science Inc., Cold Spring, New York, USA.; 13 Department of Molecular Biology, Cell Biology & Biochemistry, Brown University, Providence, Rhode Island, USA.; 14 Pulmonary, Allergy, Critical Care, and Sleep Medicine, University of Pittsburgh, Pittsburgh, Pennsylvania, USA.; 15 Dorothy P. and Richard P. Simmons Center for Interstitial Lung Disease, University of Pittsburgh School of Medicine, Pittsburgh, Pennsylvania, USA.; 16 Department of Ecology, Evolution and Organismal Biology, and The Center on the Biology of Aging, Brown University, Providence, Rhode Island, USA.

**Keywords:** Aging, Metabolism, Glucose metabolism, Insulin

## Abstract

Insulin/insulin growth factor signaling is a conserved pathway that regulates lifespan. However, long-lived loss-of-function mutants often produce insulin resistance, slow growth, and impair reproduction. Recently, a gain-of-function mutation in the kinase insert domain (KID) of the *Drosophila* insulin/IGF receptor was seen to dominantly extend lifespan without impairing insulin sensitivity, growth, or reproduction. This substitution occurs within residues conserved in mammalian insulin receptor (IR) and insulin growth factor-1 receptor (IGF-1R). We produced 2 knock-in mouse strains that carry the homologous KID Arg/Cys substitution in murine IR or IGF-1R, and we replicated these genotypes in human cells. Cells with heterodimer receptors of IR or IGF-1R induce receptor phosphorylation and phospho-Akt when stimulated with insulin or IGF. Heterodimer receptors of IR fully induce pERK, but ERK was less phosphorylated in cells with IGF-1R heterodimers. Adults with a single KID allele (producing heterodimer receptors) have normal growth and glucose regulation. At 4 months, these mice variably display hormonal markers that associate with successful aging counteraction, including elevated adiponectin and FGF21, as well as reduced leptin and IGF-1. Livers of IGF-1R females show decreased transcriptome-based biological age, which may point toward delayed aging and warrants an actual lifespan experiment. These data suggest that KID mutants may slow mammalian aging while they avoid the complications of insulin resistance.

## Abstract 2

Our novel kinase insert domain mutants of insulin receptor or insulin growth factor-1 receptor may slow mammalian aging without the complications associated with classical IlS mutants

## Introduction

Insulin/insulin growth factor signaling (IIS) is a conserved nutrient-sensing pathway that regulates lifespan in invertebrate and murine models (1–4). Loss-of-function mutations in the insulin-like receptor of *C*. *elegans* (*daf-2*) or *Drosophila* (*dInr*) can increase lifespan as much as 2-fold (5–8). In mice, lifespan can be extended nearly 50% in IGF-1R hemizygotes (+/–) in a sex- and strain-dependent manner (9). However, prolongevity mutations in these models also produce insulin resistance, slow growth, and impair reproduction (10). This paradox limits our ability to translate such results to humans: slow aging conferred by repressing insulin/IGF signaling may cause metabolic and growth-related disease.

Mature mammals have 2 cell-surface receptors that can bind insulin and IGF ligands: insulin receptor (IR) and insulin-like growth factor-1 receptor (IGF-1R) (11). The insulin and IGF-1Rs function as dimers to interact with insulin and IGF-1, from which they transduce intracellular signaling and subsequently undergo endocytosis (12, 13). Various murine mutations in these receptors have been characterized for their potential effect on aging and to document their deleterious properties. For example, mice homozygous for a mutation (IR^P1195L^), ortholog of the canonical *daf-2*(*e1370*) longevity allele of *C*. *elegans*, die soon after birth from diabetic ketoacidosis, whereas heterozygous mice have normal lifespan but developed insulin resistance with hyperinsulinemia (14, 15). Nelson et al. (16) found that mice hemizygous for a null mutation in the IR had increased lifespan in males but not in females. These mice have congenital insulin resistance, although this complication did not progress with age any faster than in WT. Disruption of IR in adipose tissue alone extends lifespan by 13% (17) and protects mice from age- and obesity-related insulin insensitivity and glucose intolerance (18). These traits, however, may be secondary to how loss of IR impairs adipose development.

Mutations of the IGF-1R also extend lifespan but, again, with some ambiguity and deleterious properties. Female but not male lifespan is increased in mice in the 129/SvPas background with 1 null allele of *Igf1r* (19). This benefit was less apparent, and sometimes became associated with glucose intolerance, when this Igf1r genotype was studied in different genetic backgrounds and in standardized laboratory conditions (9, 20). As an alternative to *Igf1r*-null mutants complemented by a WT allele, Lorenzini et al. (21) studied a hypomorph that reduced ligand *igf1* titer. There was little effect on male survival, while female survival was increased in some test locations but not in others. Neoplasm, body mass, and bone density were reduced in all of these *igf1*-deficient mice.

How insulin and IGF receptors modify aging also depends on when the manipulation is initiated. In worms, depletion of DAF-2 during early adulthood extends lifespan but associates with slowed growth, germline shrinkage, egg retention, and reduced brood size. Survival was extended without these correlated effects when DAF-2 was depleted after reproductive cessation or just in the intestine during early adulthood (22). Similarly, Mao et al. (23) tested if murine aging was slowed by monoclonal antibodies targeting the IGF-1 receptor beginning with 18-month-old mice. This improved female health span and remaining life expectancy, while reducing neoplasms and inflammation. Tissue-limited manipulations of IGF signaling can also produce age-specific effects. Increased cardiomyocyte IGF-1R signaling improves heart function in early life but accelerates cardiac aging and reduces survival in later life. In contrast, low IGF-1R signaling suppresses heart function in young mice but preserves cardiac aging (24).

*Drosophilamelanogaster*, unlike mammals, has a single insulin/IGF-like receptor (*dInr*) and 7 insulin/IGF-like peptide encoding genes (*ilps*). Genetic loss-of-function genotypes of the insulin/IGF signaling system extend lifespan but also slow growth, reduce adult size, impair fecundity and limit the ability of insulin to induce phosphorylation of AKT (pAKT) (5, 8). We recently uncovered a potentially novel mutation in the kinase insert domain (KID) (5, 25) that robustly extends lifespan without these deleterious effects (5). This Arg1466Cys substitution in the KID (denoted *dInr*^KID^ or *dInr*^353^) robustly extends lifespan in males and females while the adults are full-sized, highly fecund, and induce pAkt when heterozygous with a WT allele (*dInr*^KID^/*dInr*^wt^). Importantly, the arginine residue of the KID in *dInr* is conserved in the KIDs of mammalian IR (mouse IR at Arg1109) and IGF-1R (mouse IGF-1R at Arg1096). Here we ask how an Arg>Cys substitution within the KID of IR and IGF-1R affects young mice. We generated 2 knock-in strains that carry the KID Arg>Cys substitution in either IR (IR*^R1109C^*) or IGF-1R (IGF-1R^R1096C^). We characterize how these substitutions affect growth, neuromuscular function, metabolic health, hormonal profiles, and biological age. In a parallel human cell culture model, we ask how the mammalian KID substitutions affect ligand activation of the receptor and downstream phosphorylation of Akt and ERK. We find that heterozygote mice grow normally and are overall metabolically healthy. To varying degrees, these young mice show nascent endocrine profiles previously associated with slowed aging in several murine models. Based on a transcriptomic clock, livers of IGF-1R heterozygous females have reduced biological age and warrant an actual lifespan experiment. In ligand-stimulated cells, IGF-1R^R1096C^ and IR^R1109C^ form functional heterodimers with a WT protomer that activates pAKT but only partially activates pERK. We conclude that the tyrosine receptor KID domains of murine IR and IGF-1R are potential modulators of aging that avoid complications of receptor loss of function.

## Results

### Viable offspring with Arg>Cys substitutions in the KID of IR (InsR^R1109C^) or IGF1R (IGF-1R^R1096C^).

**Figure 1:**
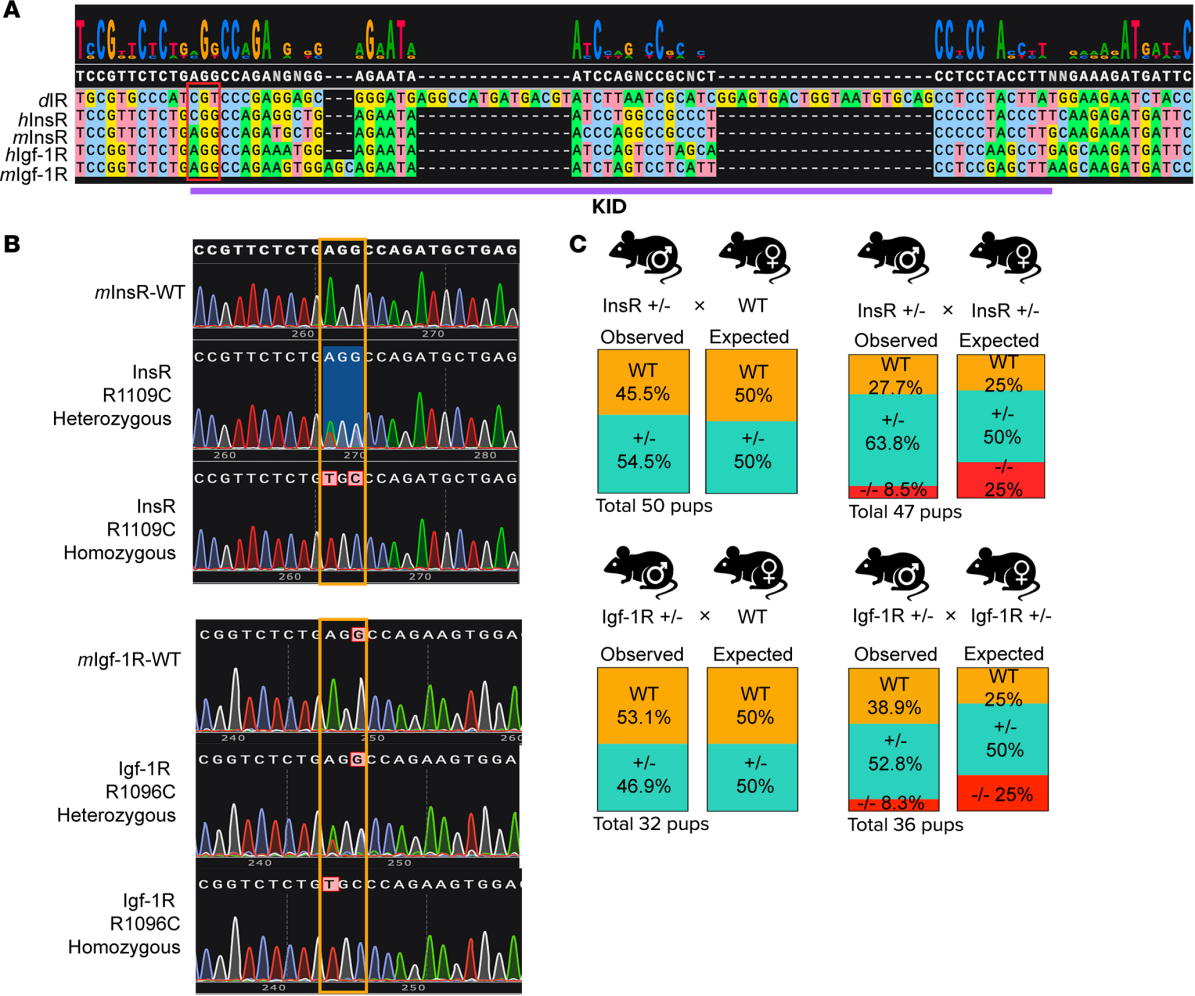
Knock-in mouse strains carrying the homologous dInR^KID^ Arg/Cys substitution in the KID of IR (InsR^R1109C^) or IGF1R (IGF-1R^R1096C^). (**A**) Sequence alignment of the KID domain for *Drosophila*, human, and mouse insulin receptor (IR) and insulin growth factor-1 receptor (IGF-1R). (**B**) DNA sequence chromatogram showing 1 peak for either AGG (WT) or TGC (homozygous) mutant and 2 peaks for heterozygous mutants. (**C**) Expected versus observed genotype ratios for offspring of WT × heterozygote and heterozygote × heterozygote.

The longevity inducing Arg>Cys substitution within the KID of dInr resides in the Arg-Pro-Glu sequence at the start of the KID (5). In general, KIDs are unstructured and variable domains that connect the N- and C-terminal lobes of receptor tyrosine kinases, including mammalian IR and IGF-1R (Figure 1A) (5, 25). We generated 2 knock-in mouse strains in the C57BL/6J background that carry the homologous KID Arg>Cys substitution in murine IR (InsR^R1109C^) or IGF-1R (IGF-1R^R1096C^) (Figure 1B). These mutations are viable as heterozygotes over a WT receptor allele. Parental crosses of WT with heterozygous InsR^R1109C^ produced F_1_ progeny as 45.5% homozygote WT and 54.5% heterozygote. Crosses of WT with heterozygous IGF-1R^R1096C^ adults produced 53.1% WT and 46.9% heterozygote offspring. Crosses of heterozygote InsR^wt^/InsR^R1109C^ parents produced F_1_ in proportions of 27.7% WT, 63.8% heterozygote, and 8.5% mutant homozygote. Offspring of 38.9% WT, 52.8% heterozygote, and 8.3% mutant homozygotes were derived from crosses of heterozygote InsR^wt^/IGF-1R^R1096C^ parents (Figure 1C). We could not generate any InsR^R1109C^, IGF-1R^R1096C^ double heterozygous pups.

### Normal growth of InsR^R1109C^ and IGF-1R^R1096C^ heterozygotes.

**Figure 2:**
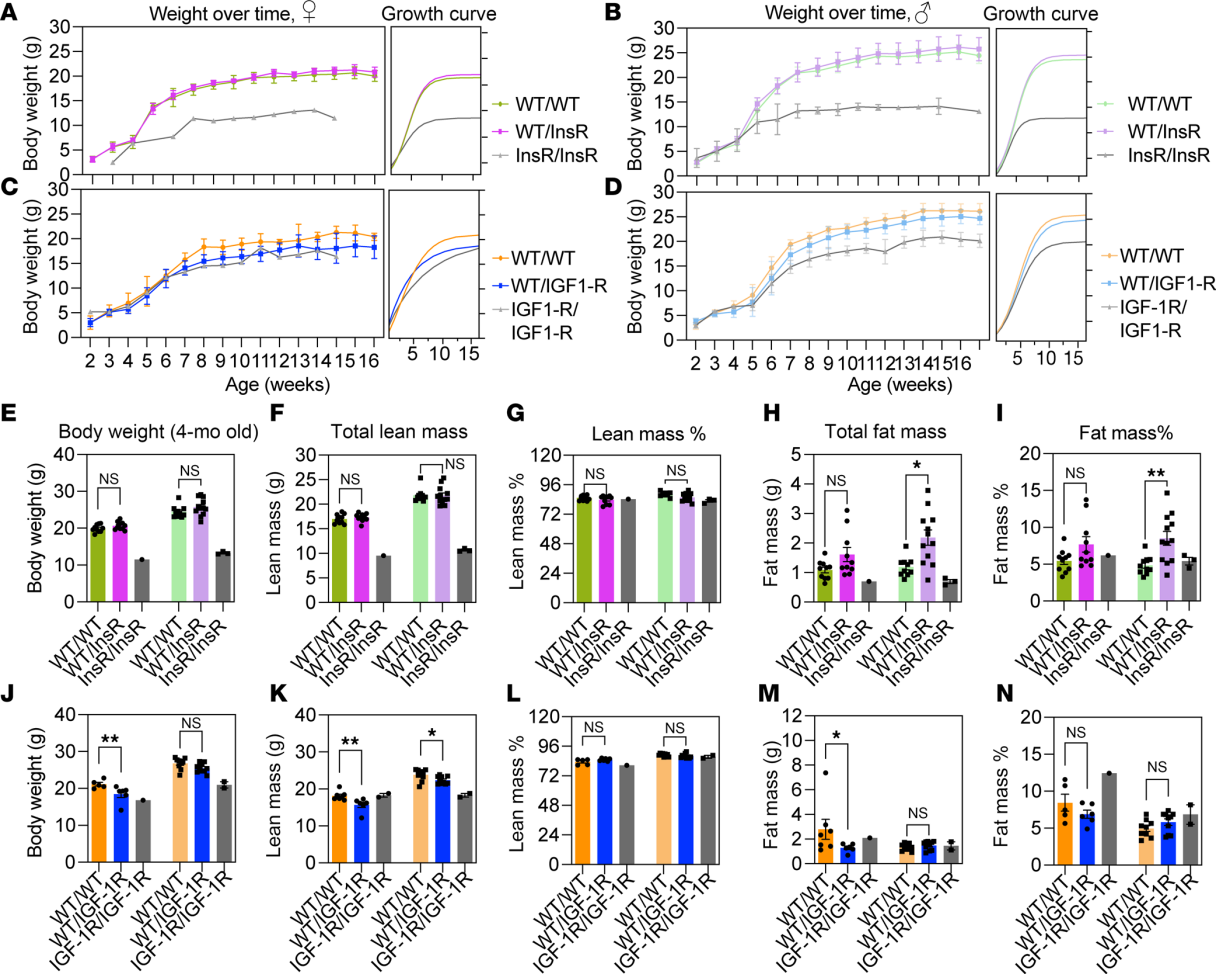
Effect of InsR^R1109C^ and IGF-1R^R1096C^ substitutions on growth rate. (**A**–**D**) Weekly mass (left panel) and logistic growth curves (right panel) in (**A**) InsR^R1109C^ female, (**B**) InsR^R1109C^ male, (**C**) IGF-1R^R1096C^ female, and (**D**) IGF-1R^R1096C^ male mice. (**E** and **J**) Female and male body weight in 4-month-old (**E**) InsR^R1109C^ and (**J**) IGF-1R^R1096C^ mice. (**F** and **K**) Female and male total lean mass in (**F**) InsR^R1109C^ and (**K**) IGF-1R^R1096C^ mice. (**G** and **L**) Female and male percent lean mass normalized to body weight in (**G**) InsR^R1109C^ and (**L**) IGF-1R^R1096C^ mice. (**H** and **M**) Female and male total fat mass weight in (**H**) InsR^R1109C^ and (**M**) IGF-1R^R1096C^ mice. (**I** and **N**) Female and male percent fat mass normalized to body weight in (**I**) InsR^R1109C^ and (**N**) IGF-1R^R1096C^ mice. WT and heterozygotes were available for statistical comparison (females WT/WT and WT/InsR *n* = 10; males WT/WT *n* = 10 and WT/InsR *n* = 12; females WT/WT *n* = 5 and WT/IGF-1R *n* = 6; males WT/WT *n* = 9 and WT/IGF-1R *n* = 10). Homozygote mutants were rare. Data are shown as mean ± SD, Student’s *t* test. *P < 0.05; **P < 0.01.

In *Drosophila*, growth is not retarded in WT/KID heterozygotes (dInR^+^/dInR^KID^) (5). We therefore assessed how growth through 4 months of age was affected when mice were heterozygous or homozygous for InsR^R1109C^ or IGF-1R^R1096C^ (Figure 2, A–D). Because we derived few F_1_ homozygotes, hereafter, we include those observations without statistical analyses. InsR^+^/InsR^R1109C^ heterozygotes gained the same mass as WT siblings, while mutant homozygotes at first gained the same mass as WT but plateaued at a lighter weight (38% decrease in females, 48% decrease in males) (Figure 2E). Estimated growth rate based on a parametric model determined the rates were similar among WT and InsR^R1109C^ heterozygotes (Figure 2, A, B, and E).

Female IGF-1R^R1096C^ heterozygotes accumulated less mass than WT at 4 months (12.7% decrease); male heterozygotes weighed as much as WT (Figure 2, C, D, and J). The mass of IGF-1R^R1096C^ homozygotes was reduced in both sexes (Figure 2, C, D, and J). The growth rate of IGF-1R^R1096C^ heterozygotes equaled that of WT in males but was 0.63-fold reduced in females.

We used NMR to measure how the genotypes affected body composition. InsR^R1109C^ and IGF-1R^R1096C^ heterozygotes had the same percentage lean mass as WT (Figure 2, G and L). Total lean mass was slightly reduced in IGF-1R^R1096C^ heterozygotes of both sexes (Figure 2, F and K). Fat mass (total and percentage) was moderately increased in male InsR^R1109C^ heterozygotes (Figure 2, H and I), while total fat was reduced in female IGF-1R^R1096C^ heterozygotes (Figure 2, M and N). Overall, heterozygotes with an Arg>Cys substitution in the KID of murine IR and IGF1R show few or modest effects on growth.

### Normal metabolic rates in heterozygote InsR^R1109C^ and IGF-1R^R1096C^.

**Figure 3:**
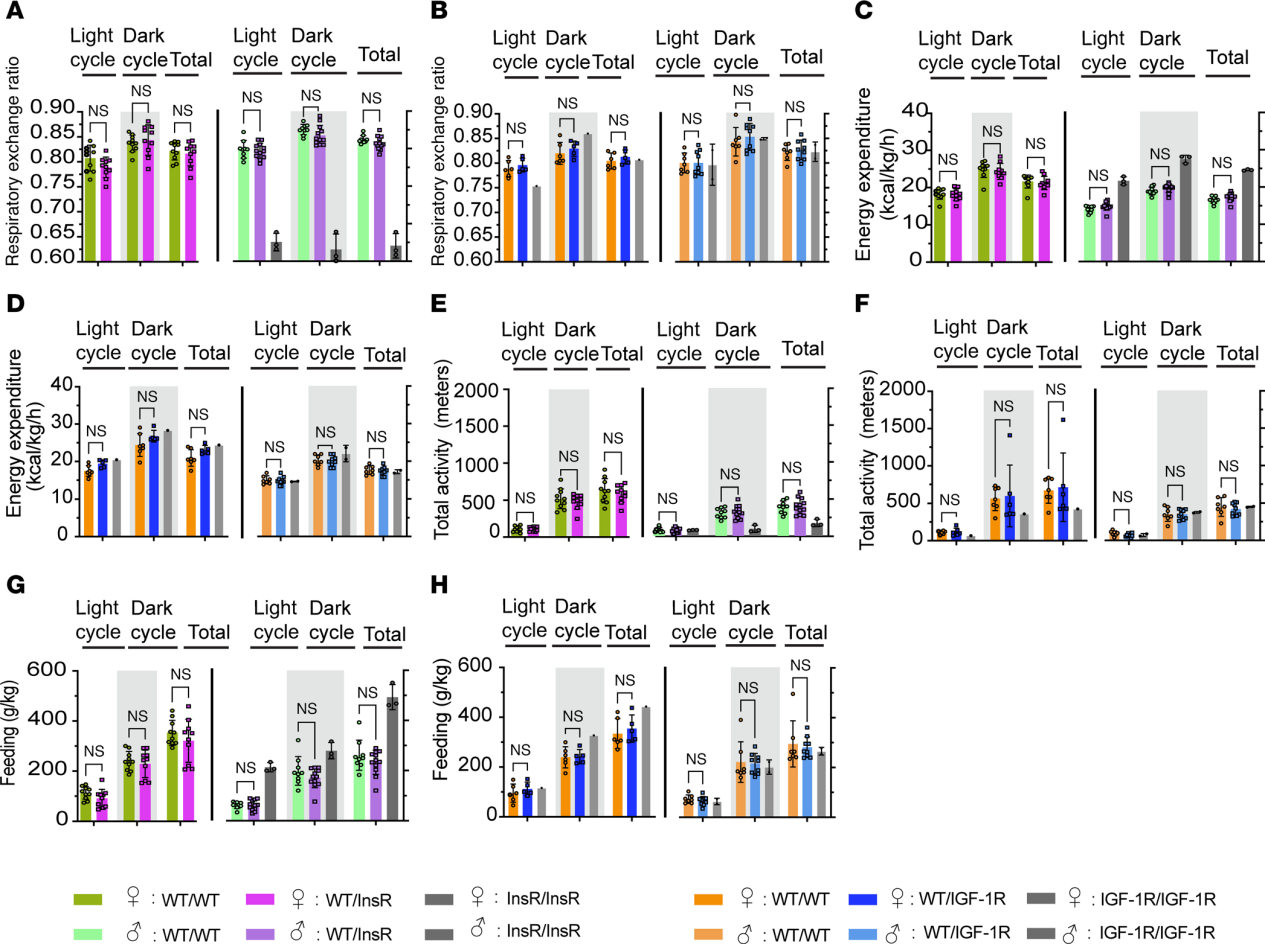
Metabolic rate of InsR^R1109C^ and IGF-1R^R1096C^ heterozygous mice. (**A** and **B**) Respiratory exchange ratio (RER) in (**A**) InsR^R1109C^ and (**B**) IGF-1R^R1096C^ mice, 24-hour average (per kg of lean mass). (**C** and **D**) Energy expenditure (EE), 24 hr average (per kg of lean mass) in (**C**) InsR^R1109C^ and (**D**) IGF-1R^R1096C^. (**E** and **F**) Total activity 24-hour average in (**E**) InsR^R1109C^ and (**F**) IGF-1R^R1096C^. (**G** and **H**) Feeding, 24-hour average (per kg of lean mass) in (**G**) InsR^R1109C^ and (**H**) IGF-1R^R1096C^. WT and heterozygous mice were available for statistical comparison (females WT/WT and WT/InsR *n* = 10; males WT/WT *n* = 10 and WT/InsR *n* = 12; females WT/WT *n* = 5 and WT/IGF-1R *n* = 6; males WT/WT *n* = 9 and WT/IGF-1R *n* = 10). Homozygous mutant mice were rare. Data are shown as mean ± SD, Student’s *t* test.

We used indirect calorimetry to evaluate how InsR^R1109C^ and IGF-1R^R1096C^ affect whole animal metabolism by measuring energy expenditure (EE), respiratory exchange ratio (RER), total activity, and food uptake in mice at 4 months of age. RER describes the substrates used to generate energy (26–30). The RER of WT heterozygotes with InsR^R1109C^ or IGF-1R^R1096C^ was like that of WT in both sexes (0.8–0.85), indicative of substrates from a mixture of protein, lipid, and carbohydrate (Figure 3, A and B, and Supplemental Figure 1, A–D; supplemental material available online with this article; https://doi.org/10.1172/jci.insight.189683DS1). RER was reduced (<0.7) among the available male InsR^R1109C^ homozygotes, indicating these animals used lipids rather than carbohydrates to produce energy as occurs when cells are insulin resistant (31) (Figure 3A and Supplemental Figure 1B). Resting EE (Figure 3, C and D, and Supplemental Figure 1, E–H) total activity (Figure 3, E and F, and Supplemental Figure 1, I–L), and food intake (Figure 3, G and H, and Supplemental Figure 1, M–P) did not differ among WT and InsR^R1109C^ or IGF-1R^R1096C^ heterozygote siblings. Overall, InsR^R1109C^ and IGF-1R^R1096C^ heterozygotes present metabolic profiles indistinguishable from their WT littermates.

### Normal glycated hemoglobin in heterozygote InsR^R1109C^ and IGF-1R^R1096C^.

**Figure 4:**
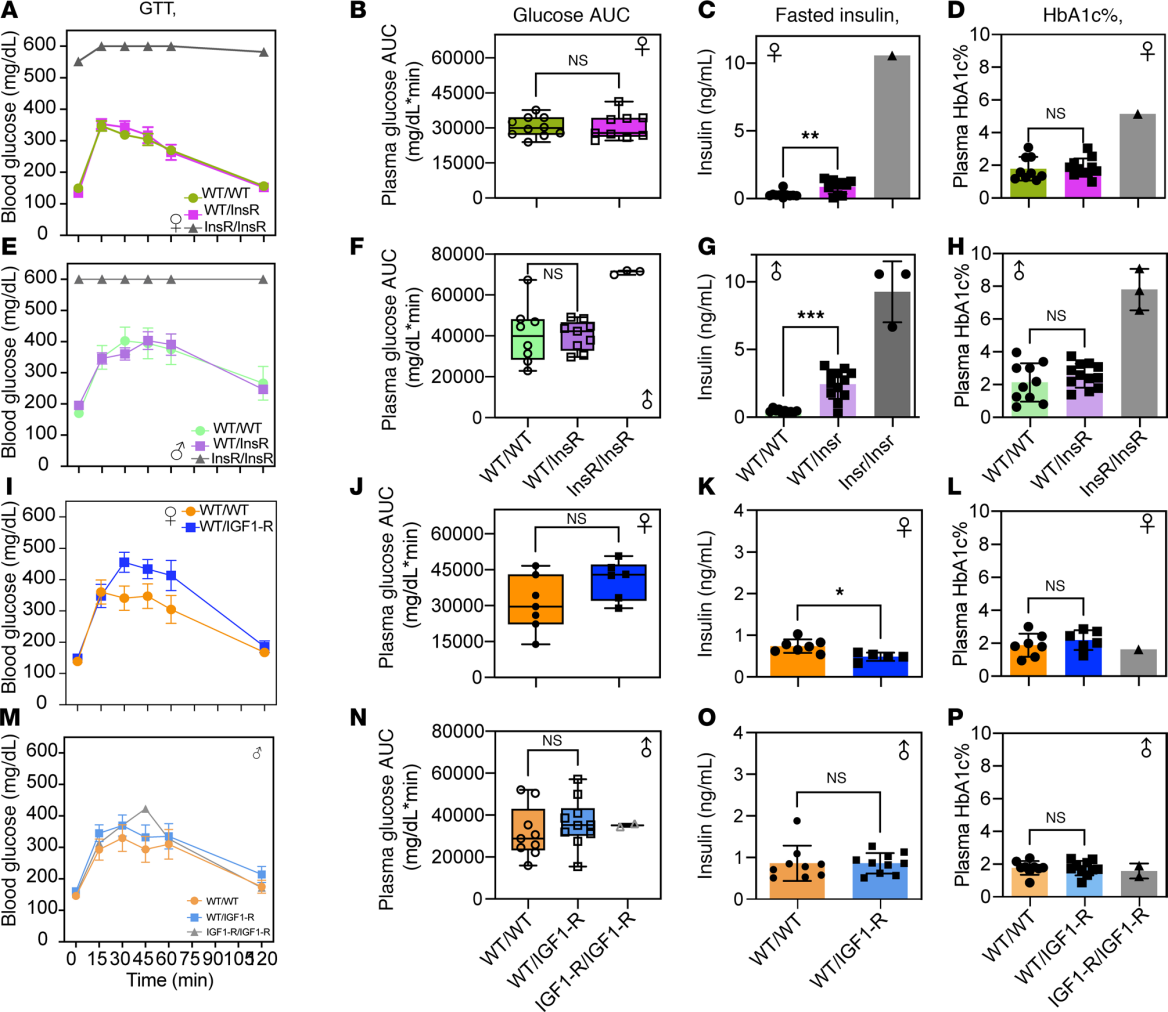
Glycated hemoglobin in InsR^R1109C^ and IGF-1R^R1096C^ heterozygous mice. (**A**, **E**, **I**, and **M**) Blood glucose level measured during glucose tolerance test (GTT) using 1.5g/kg glucose dose in (**A**) female InsR^R1109C^, (**E**) male InsR^R1109C^, (**I**) female IGF-1R^R1096C^, and (**M**) male IGF-1R^R1096C^. (**B**, **F**, **J**, and **N**) Area under the curve (AUC) for glucose in (**B**) female InsR^R1109C^, (**F**) male InsR^R1109C^, (**J**) female IGF-1R^R1096C^, and (**N**) male IGF-1R^R1096C^. (**C**, **G**, **K**, and **O**) Fasted plasma insulin levels in (**C**) female InsR^R1109C^, (**G**) male InsR^R1109C^, (**K**) female IGF-1R^R1096C^, and (**O**) male IGF-1R^R1096C^. (**D**, **H**, **L**, and **P**) Glycated hemoglobin A1C percentage in (**D**) female InsR^R1109C^, (**H**) male InsR^R1109C^, (**L**) female IGF-1R^R1096C^, and (**P**) male IGF-1R^R1096C^. WT and heterozygous were available for statistical comparison (females WT/WT *n* = 9–10 and WT/InsR *n* = 9–11; males WT/WT *n* = 8–10 and WT/InsR *n* = 9–12; females WT/WT *n* = 7 and WT/IGF-1R *n* = 6; males WT/WT *n* = 9–10 and WT/IGF-1R *n* = 10–11). Homozygous mutant mice were rare. Data are shown as mean ± SD, Student’s *t* test. * *P* < 0.05; **P < 0.01; ***P < 0.001.

To assess glucose homeostasis in InsR^R1109C^ and IGF-1R^R1096C^ heterozygotes, we measured glucose tolerance, plasma insulin, and glycated hemoglobin. Plasma glucose and glycated hemoglobin were normal in male and female InsR^R1109C^ heterozygotes, although mice within the glucose tolerance test (GTT) had elevated fasting insulin (Figure 4, A–H, and Supplemental Figure 2, A–D). As expected, available homozygotes presented elevated plasma glucose, insulin, and glycated hemoglobin, consistent with a state of insulin resistance (Figure 4, A–H, and Supplemental Figure 2, A–D). Male and female IGF-1R^R1096C^ heterozygotes presented normal glucose tolerance (GTT), glycated hemoglobin, and insulin titer except for fasted females where plasma insulin was reduced relative to WT (Figure 4, I–P, and Supplemental Figure 2, E–H).

Overall, when heterozygous, the murine KID mutation in InsR^R1109C^ results in moderate, compensatory hyperinsulinemia, while a heterozygote with the KID mutation IGF-1R^R1096C^ has little effect on energy metabolism and glucose homeostasis.

### Improved neuromuscular function in heterozygotes of InsR^R1109C^ and IGF-1R^R1096C^.

**Figure 5:**
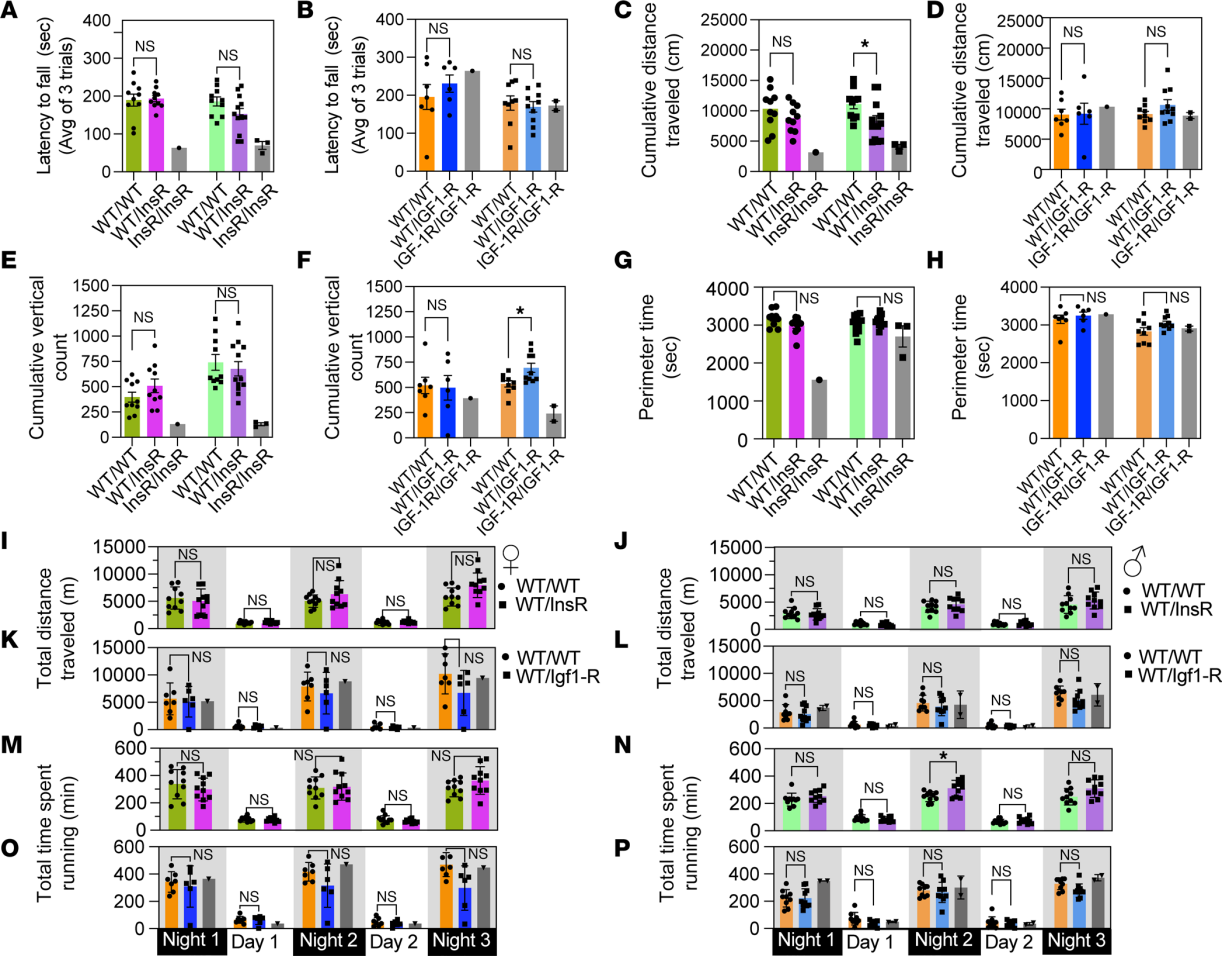
Neuromuscular function in InsR*^R1109C^* and IGF-1R*^R1096C^* heterozygous mice. (**A** and **B**) Latency to rotarod test fall in female and male (**A**) InsR^R1109C^ and (**B**) IGF-1R^R1096C^. (**C** and **D**) Cumulative distance traveled in the open field test in female and male (**C**) InsR^R1109C^ and (**D**) IGF-1R^R1096C^. (**E** and **F**) Cumulative Vertical Activity the open field test in female and male (**E**) InsR^R1109C^ and (**F**) IGF-1R^R1096C^. (**G** and **H**) Time spent in margin areas in the open field test in female and male (**G**) InsR^R1109C^ and (**H**) IGF-1R^R1096C^. (**I**, **J**, **K**, and **L**) Total distance traveled in wheel-running in (**I**) female InsR^R1109C^, (**J**) male InsR^R1109C^, (**K**) female IGF-1R^R1096C^, and (**L**) male IGF-1R^R1096C^. (**M**, **N**, **O**, and **P**) Total time spent wheel-running in (**M**) female InsR^R1109C^, (**N**) male InsR^R1109C^, (**O**) female IGF-1R^R1096C^, and (**P**) male IGF-1R^R1096C^. WT and heterozygous were available for statistical comparison (females WT/WT and WT/InsR *n* = 10; males WT/WT *n* = 10 and WT/InsR *n* = 10–12; females WT/WT *n* = 7 and WT/IGF-1R *n* = 6; males WT/WT *n* = 9 and WT/IGF-1R *n* = 9–10). Homozygous mutant mice were rare. Data are represented as means ± SD, Student’s *t* test. * *P* < 0.05.

We assessed neuromuscular function of young mutant mice through rotarod agility, open field exploratory activity, and spontaneous wheel running. The ability to maintain balance on the accelerating rotarod did not differ between InsR^R1109C^ or IGF-1R^R1096C^ heterozygotes relative to their age- and sex-matched littermate WT controls (Figure 5, A and B). Motor coordination was intact in homozygous IGF-1R mice relative to heterozygotes and WT, although InsR homozygous appeared to be strongly impaired (based on our limited sample size) (Figure 5, A and B). In the open field test, which measures spontaneous exploratory activity, IGF-1R^R1096C^ heterozygotes traveled the same distance as age- and sex-matched WT littermates. Interestingly, female InsR^R1109C^ heterozygotes but not males traveled less than age- and sex-matched WT littermates (Figure 5, C and D). Rearing behavior, a typical exploratory behavior in mice that is measured by cumulative vertical activity in the open field, was increased in IGF-1R^R1096C^ male heterozygotes but not female IGF-1R^R1096C^ heterozygotes relative to age- and sex-matched WT littermate controls (Figure 5F). Cumulative vertical activity in the open field was unaltered in InsR^R1109C^ heterozygotes relative to age- and sex-matched WT littermate controls (Figure 5E). Thigmotaxis behavior, measured by time spent at the perimeter of the open field, is an indicator of anxiety-like behavior and was similar across all sexes and genotypes (Figure 5, G and H). InsR^R1109C^ and IGF-1R^R1096C^ homozygotes had reduced cumulative distance traveled and rearing (based on limited observations) (Figure 5, C–F). Voluntary home cage wheel running assesses spontaneous physical activity. As expected, all patients were significantly more active during the dark cycle relative to the light cycle. InsR^R1109C^ heterozygote males and females ran the same total distance. Male and female InsR^R1109C^ heterozygotes were similar to WT with the exception of time spent running on the second night in males (Figure 5, I, J, M, and N). IGF-1R^R1096C^ heterozygotes and WTs were similar in both total distance and time spent running (Figure 5, K, L, O, and P).

Overall, young InsR^R1109C^ heterozygotes perform slightly better as males in wheel running (males) but less so as females. IGF-1R^R1096C^ heterozygote males have modestly elevated activity in open field as measured by rearing behavior.

### Aging-associated hormones of InsR^R1109C^ and IGF-1R^R1096C^ heterozygotes.

We measured plasma titers of FGF21, IGF-1, adiponectin, leptin, and GDF15 to assess how InsR^R1109C^ and IGF-1R^R1096C^ affect hormones that are sometimes associated with successful aging. We quantified plasma cholesterol and triglycerides to assess metabolic health. In all assays, plasma was collected from 4-month-old mice in the morning after 6 hours fasting.

**Figure 6:**
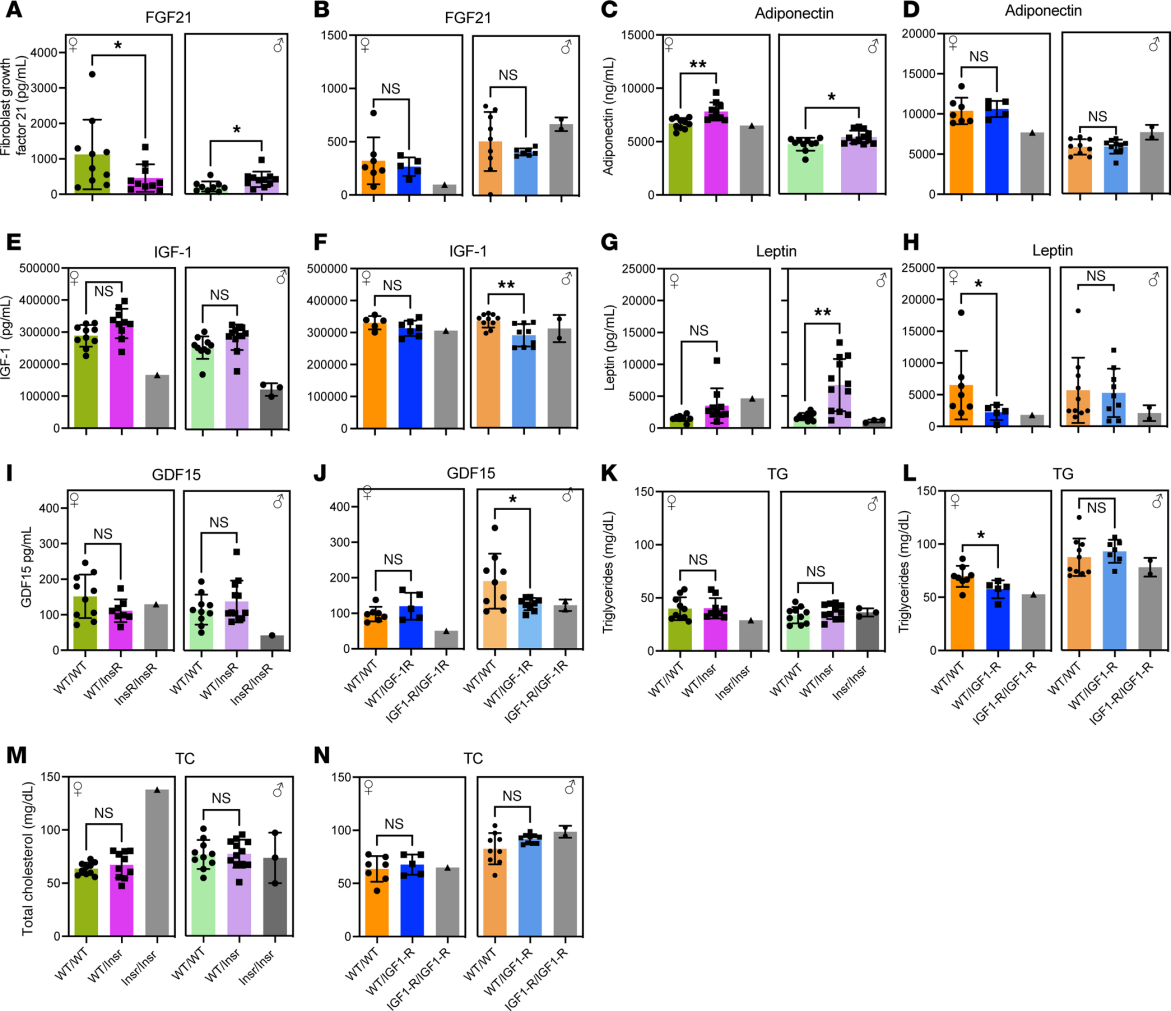
Hormonal and metabolite profiles in InsR*^R1109C^* and IGF-1R^R1096C^ heterozygous mice. (**A**–**L**) Hormone plasma levels in female and male mice from InsR^R1109C^ and IGF-1R^R1096C^ mice respectively, of FGF21 (**A** and **B**), Adiponectin (**C** and **D**), IGF-1 (**E** and **F**), Leptin (**G** and **H**), GDF15 (**I** and **J**), Triglycerides (TG) (**K** and **L**), and Total cholesterol (TC) (**M** and **N**). WT and heterozygous were available for statistical comparison (females WT/WT and WT/InsR *n* = 10; males WT/WT *n* = 9–10 and WT/InsR *n* = 11–12; females WT/WT *n* = 7 and WT/IGF-1R *n* = 5–6; males WT/WT *n* = 10 and WT/IGF-1R *n* = 9–10). Homozygous mutant mice were rare. Data are shown as mean ± SD, Student’s *t* test. *P < 0.05; **P < 0.01.

FGF21 was increased in heterozygote InsR^R1109C^ males but decreased in females, while FGF21 titer in IGF-1R^R1096C^ heterozygous mice was equal to that of WT (Figure 6, A and B). Adiponectin was increased in both male and female InsR^R1109C^ heterozygotes but did not differ between WT and heterozygotes of IGF-1R^R1096C^ (Figure 6, C and D). IGF-1 levels were not different among InsR^R1109C^ heterozygotes and WTs in either sex, but the hormone was significantly decreased in IGF-1R^R1096C^ male heterozygotes (Figure 6, E and F). The satiety hormone leptin was elevated in male InsR^R1109C^ heterozygotes, even though these mice have normal weight and food intake. In contrast, leptin was significantly decreased in IGF-1R^R1096C^ female heterozygotes but not in males (Figure 6, G and H). The titer of GDF15 typically increases with chronological age and stress in WT mice (32–34). Here, the titer at 4 months was less in IGF-1R^R1096C^ heterozygote females than in WT but not in males. GDF15 did not significantly differ between InsR^R1109C^ heterozygotes and WT (Figure 6, I and J). Plasma triglycerides were significantly decreased in IGF-1R^R1096C^ female heterozygotes but not in males or in InsR^R1109C^ heterozygotes (Figure 6, K and L). We found no significant differences in plasma total cholesterol among genotype or sex (Figure 6, M and N).

Overall, young InsR^R1109C^ and IGF-1R^R1096C^ heterozygous adults present some hormonal changes that are seen in established models of retarded murine aging (elevated FGF21, elevated adiponectin, decreased IGF-1). These patterns, however, are quite variable among genotypes and sex dependent, as seen for elevated FGF21 and leptin in InsR^R1109C^ males as well as reduced GDF15 and plasma triglycerides in IGF-1R^R1096C^ females.

### InsR^R1109C^ and IGF-1R^R1096C^ heterodimer receptors have partial altered function.

We determined how the KID mutation in IR or IGF-1R affects receptor function, measured as ligand-stimulated receptor kinase activity in human cell culture and by downstream signaling in murine tissue and in human cell culture.

**Figure 7:**
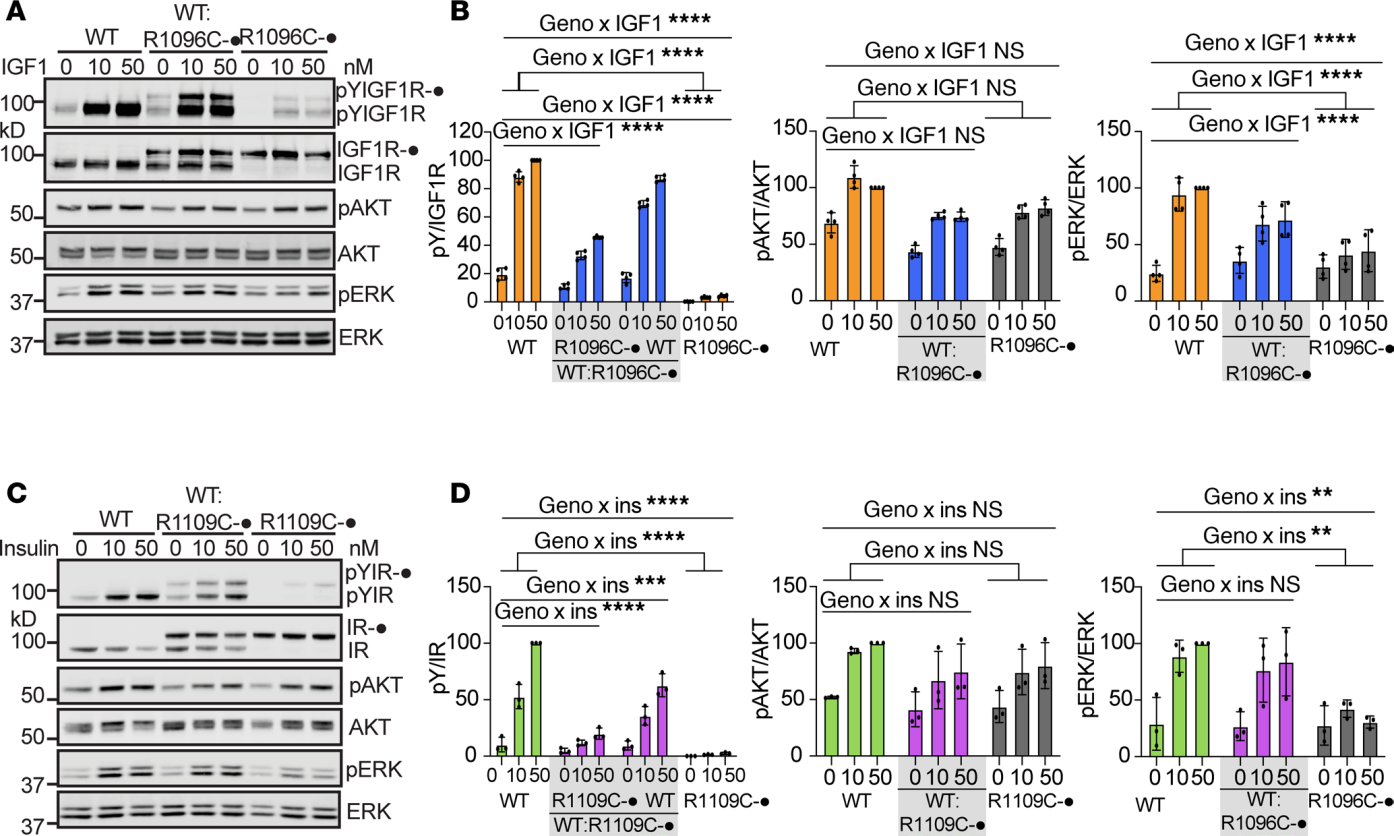
Receptor function in transfected 293FT cells with IGF-1R WT:R1096C heterodimers and IR WT:R1109C heterodimers. (**A**) IGF-1R signaling in response to the indicated concentrations of IGF-1 for 10 minutes in IR/IGF-1R knockout 293FT cells expressing IGF-1R WT and mutant receptors. (**B**) Quantitative analysis of the Western blot data. Levels of IGF-1R autophosphorylation were normalized to total IGF-1R levels and presented as intensities relative to those in IGF-1R WT cells treated with 50 nM IGF1. Data are shown as mean ± SD. Significance evaluated by 2-way ANOVA with interaction among *n* = 4 independent experiments, ***P < 0.0001. (**C**) IR signaling in response to concentrations of insulin for 10 minutes in IR/IGF-1R knockout 293FT cells expressing IR WT and mutant receptors. (**D**) Quantitative analysis of the Western blot data. Levels of IR autophosphorylation were normalized to total IR levels and presented as intensities relative to those in IR WT cells treated with 50 nM insulin. Data are shown as mean ± SD. Significance evaluated by 2-way ANOVA with interaction among *n* = 3 independent experiments, **P < 0.01; ***P < 0.001; ****P < 0.0001.

We transfected IR/IGF-1R double-knockout 293FT cells with human IGF-1R^WT^ alone, IGF-1R^R1095C^ alone (corresponding to mouse IGF-1R R1096C, hereafter referred to as R1096C), or a 1:1 mixture of both plasmids to produce heterodimer receptors (Figure 7, A and B). To assess relative expression of these receptor types, we cotransfected Myc-tagged R1096C with untagged IGF-1R^WT^. IGF-1 stimulation robustly induced autophosphorylation of IGF-1R^WT^ and phosphorylation of AKT and ERK. In contrast, R1096C homodimers exhibited markedly reduced autophosphorylation. Despite this defect, IGF-1 ligand still induced AKT phosphorylation to approximately 80% of the level observed in WT-expressing cells, whereas there is only weak phosphorylation of ERK (Figure 7, A and B). Assuming equal expression, cotransfection of IGF-1R^WT^ and R1096C is expected to generate 25% WT:WT homodimers, 50% WT:R1096C heterodimers, and 25% R1096C:R1096C homodimers. If the heterodimer is inactive, IGF-1–induced autophosphorylation should resemble that of cells expressing R1096C alone. However, cells coexpressing WT and R1096C showed IGF-1R autophosphorylation at ~50% of WT levels and pERK activation at ~70% of those in WT-expressing cells, suggesting that the WT:R1096C heterodimer produces partial functional activity (Figure 7, A and B).

We performed similar experiments using human IR^WT^ and an IR^R1107C^ mutant (corresponding to mouse IR-A R1109C, hereafter referred to as R1109C) (Figure 7, C and D). R1109C homodimers showed greatly reduced autophosphorylation and impaired pERK, while pAKT remained comparable with IR^WT^ homodimer cells. Coexpression of IR^WT^ significantly restored autophosphorylation and pERK signaling in the presence of R1109C. Together, these results support the conclusion that both IGF-1R WT:R1096C and IR WT:R1109C heterodimers are functional whereby they retain kinase activity and partial ability to engage downstream signaling pathways.

We likewise assessed signal transduction pathways (pERK/ERK and pAKT/AKT) from liver tissue of InsR^R1109C^ and IGF-1R^R1096C^ heterozygote mice. We also measured pACC/ACC and pS6/S6 to describe altered AMPK and mTORC1 activity, which are characteristic of some long-lived mice (35–39).

pErk/Erk levels were significantly higher in livers of male and female InsR^R1109C^ heterozygotes (Supplemental Figure 3, A–D) but were not affected in IGF-1R^R1096C^ heterozygotes (Supplemental Figure 3, A–D). pACC/ACC levels were normal in InsR^R1109C^ females but significantly decreased in livers of male InsR^R1109C^ heterozygotes (Supplemental Figure 3, A–D). In contrast, pACC/ACC was elevated in male and female IGF-1R^R1096C^ heterozygotes (Supplemental Figure 3, A–D). pAKT/AKT was normal in InsR^R1109C^ females and males (Supplemental Figure 3, A–D) but significantly decreased in IGF-1R^R1096C^ females and increased in males (Supplemental Figure 3, A–D); pS6/S6 was significantly lower in InsR^R1109C^ females but normal in InsR^R1109C^ males (Supplemental Figure 3, A–D). pS6/S6 was reduced in female IGF-1R^R1096C^ heterozygotes but was upregulated in male IGF-1R^R1096C^ heterozygotes (Supplemental Figure 3, A–D).

Overall, the activity of TOR, AMPK, and MAPK signaling in mouse tissue was partially altered by InsR^R1109C^ and IGF-1R^R1096C^ when these mutants complement a WT receptor allele. Some of these changes (Table 1) are seen in other models of extended murine longevity, while other observations here are ambiguous, such as where male IGF-1R^R1096C^ increase both AMPK activity and TOR activity.

### Decreased biological age in livers of heterozygous female IGF-1R^R1096C^ mice.

In this preliminary report, we cannot assess aging through survival analysis. Accordingly, we use transcriptomic biomarkers as proxies of aging and mortality. Recently developed transcriptome-based clocks can predict age-associated mortality and identify the molecular pathways that mediate differences in biological age (40).

**Figure 8:**
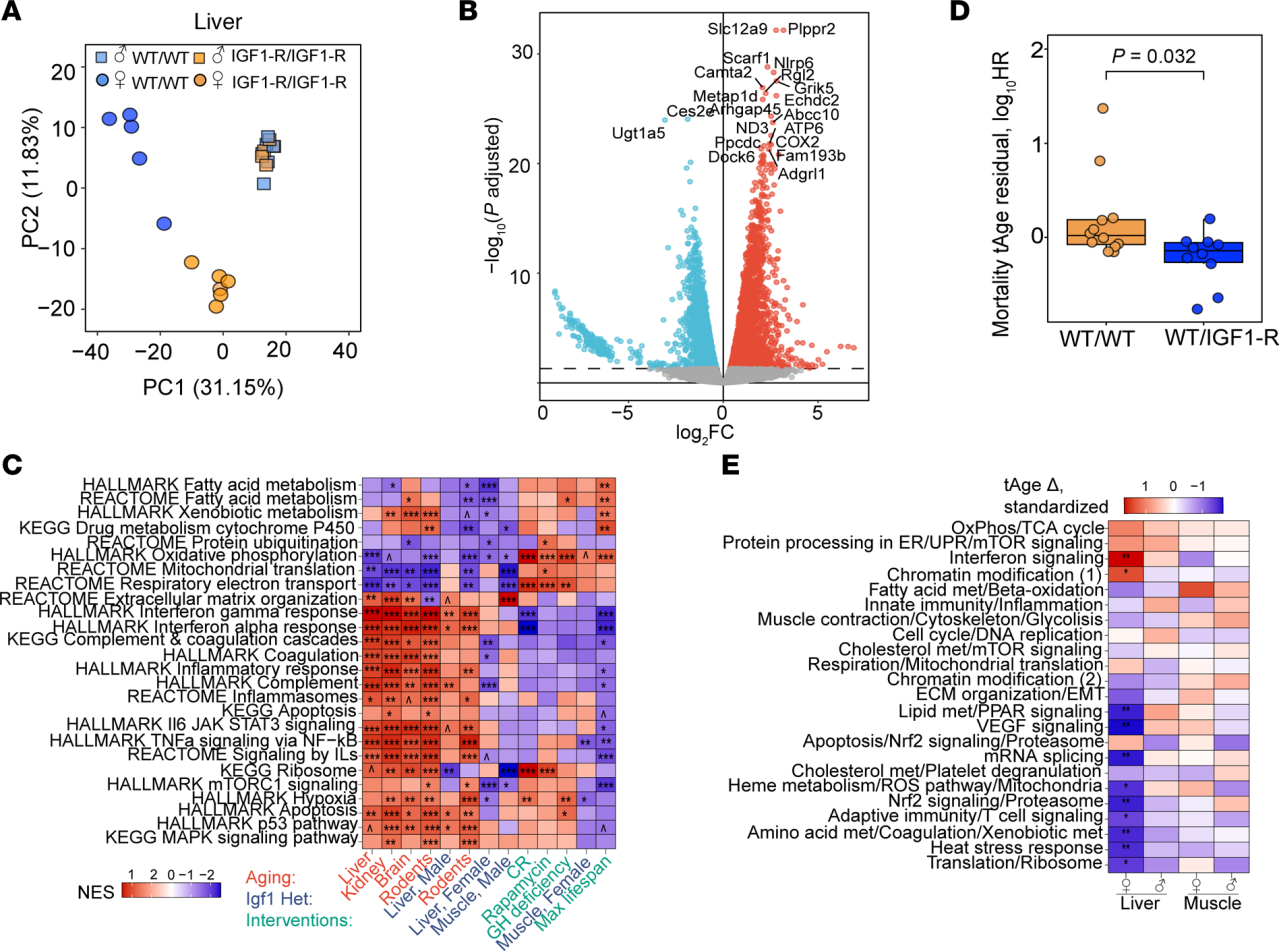
Transcriptome-estimated biological age in liver of female IGF-1R^R1096C^ heterozygote. (**A**) Principal component analysis (PCA) of liver gene expression from WT and IGF-1R^R1096C^ heterozygous mice. (**B**) Volcano plots of gene expression changes induced in the livers of IGF-1R^R1096C^ heterozygous female mice compared with WT. Benjamini-Hochberg (BH) *P*_adj_ value threshold at 0.05 (dotted line). (**C**) Gene set enrichment analysis (GSEA) of transcriptomic changes induced in IGF-1R^R1096C^ heterozygous mice relative to WT (blue), signatures of aging and mortality (red), and biomarkers of lifespan-extending interventions (green). Gene sets derived from KEGG, REACTOME, and HALLMARKS ontologies (full data in Supplemental Table 2). NES: normalized enrichment score. (**D**) Mortality transcriptomic age (tAge) of WT and IGF-1R^R1096C^ heterozygous female mice pooled across skeletal muscle and liver, as assessed with the rodent multi-tissue Elastic Net (EN) clock. tAges were adjusted for tissue type using an ANOVA model, and the resulting residuals are shown. Group differences were assessed using ANOVA, with BH *P*_adj_ values. (**E**) Standardized change in mortality tAge in IGF-1R^R1096C^ heterozygous mice relative to sex-matched WT controls, assessed using module-specific transcriptomic clocks of expected mortality. OxPhos, Oxidative Phosphorylation; TCA, Tricarboxylic Acid; ER, Endoplasmic reticulum; UPR, Unfolded Protein Response; met, metabolism; ECM, Extracellular Matrix; EMT, Epithelial-mesenchymal transition. ^*P*_adj_ < 0.1, *P_adj_ < 0.05, **P_adj_ < 0.01, ***P_adj_ < 0.001.

We focused on IGF-1R mice using RNA-Seq from gastrocnemius muscle and liver of WT and heterozygote IGF-1R^R1096C^ of both sexes at 4 months of age. We did not include heterozygote InsR^R1109C^ mice in the analysis because they develop mild hyperinsulinemia. PCA of muscle samples separated the samples based on sex but not by genotype (Supplemental Figure 4A). PCA of the liver tissue separated samples by sex on the first principal component and by genotype among females on the second principal axis (Figure 8A). This suggests there is a sex-specific effect of the IGF-1R^R1096C^ KID within the liver. Accordingly, over 5,000 differentially expressed genes (*P*_adj_ < 0.05) were identified in livers of IGF-1R^R1096C^ heterozygous females, while few DEGs were seen in female muscle or in either tissue of males (Figure 8B, Supplemental Figure 4B, and Supplemental Table 1). Interestingly, the expression of *Igf1* and *Igf1r* were significantly upregulated in the liver of female IGF-1R^R1096C^ mice, while IGF-1 in plasma was significantly decreased in IGF-1R^R1096C^ male heterozygotes (Supplemental Figure 4C).

We compared the transcriptional profiles from the female IGF-1R^R1096C^ liver to characteristic signatures seen in established murine models of delayed aging (41, 42). This revealed a positive correlation with longevity-associated signatures and a negative correlation with aging- and mortality-associated biomarkers. We observed a positive correlation with the signature specific to calorie restriction (*P*_adj_ < 0.001) and a negative association with signatures characteristic of aging-associated degeneration (kidney aging and rodent aging, each *P*_adj_ < 0.001) (Supplemental Figure 4D). Gene set enrichment analysis (GSEA) from IGF-1R^R1096C^ liver samples revealed a negative normalized enrichment score (NES) for metabolic processes (fatty acid metabolism, oxidative phosphorylation), complement, mTORC1 signaling, and hypoxia (Figure 8C and Supplemental Table 2). The observed downregulation of mTORC1-associated genes is consistent with our Western blot data where pS6 is decreased in IGF-1R^R1096C^ livers (Supplemental Figure 3C).

We then applied multitissue transcriptomic clocks of expected mortality (40) to estimate transcriptomic age (tAge) in tissue samples of female WT and IGF-1R^R1096C^ heterozygous animals. While individual organs did not show statistically significant differences, likely due to low sample size (Supplemental Figure 4E), female IGF-1R^R1096C^ heterozygotes displayed significantly lower tAge when liver and muscle tissues were pooled (Figure 8D). We applied module-specific transcriptomic mortality clocks to gain mechanistic insight into the biological pathways contributing to this effect (40). Several transcriptomic modules showed reduced tAge in liver tissue of IGF-1R^R1096C^ females (*P*_adj_ < 0.05) (Figure 8D), but no significant differences were observed in other tissues or in males. Strongly affected modules included lipid metabolism, VEGF signaling, mRNA splicing, mitochondria, NRF2 signaling, adaptive immunity, amino acid metabolism, heat-stress response, and translation (Figure 8E). Modules related to IFN signaling and chromatin modification showed modest increases in tAge, suggesting that while antiaging effects are predominant in this model, some proaging signals may be present.

In summary, female IGF-1R^R1096C^ heterozygous mice exhibit decreased biological age, particularly in the liver, highlighting potential molecular pathways contributing to the health-promoting effect of this genetic model, and this finding warrants an actual lifespan experiment.

## Discussion

### Mouse InsR^R1109C^ or IGF-1R^R1096C^ substitutions in the KID.

A mutation in the KID of the *Drosophila* insulin/IGF receptor extends lifespan without impairing insulin-sensitivity, growth, and reproduction (5). The allele (referred to as *dInr*^KID^ or *dInr*^353^) contains an amino acid substitution (Arg1466Cys) within a sequence of the KID that is conserved in the KID of the mammalian insulin receptor (IR-A; Arg1107 in human, Arg1109 in mouse) and the IGF-1R (Arg1095 in human, Arg1096 in mouse) (Supplemental Table 3). This receptor domain has previously been known only for its effect on pathology. Donohue syndrome (OMIM #246200) is a rare and severe autosomal recessive disease caused by mutations in the insulin receptor. Homozygotes have strong insulin resistance, severe growth retardation, and hypertrichosis (43). Donohue syndrome can be caused by a homozygous Arg1107Gln substitution in the insulin receptor KID (44), the residue mutated in the long-lived of *Drosophila* (Arg1466Cys). Heterozygote parents have mild type A insulin resistance syndrome (43, 44). In recent cell-based studies, Chen et al. (45) showed Arg1104Glu combined with Arg1107Glu inhibits A-loop-tyrosine phosphorylation in stimulated mutant IR homodimers. Hall et al. (46) investigated how human IR substitutions induce premature endocytosis, finding that homodimers with the Arg1107Gln substitution remain at the plasma membrane in cells at their basal, unstimulated state. No data yet describe how substituting nonpolar cysteine for arginine in the IR KID will affect cell traits or animal phenotypes, but we expect it will not recapitulate what is seen with substitution with polar Gln or negatively charged Glu.

To empirically explore the biological relevance of Arg>Cys in the mammalian receptors, we generated 2 knock-in mouse models that carry the homologous fly substitution (Arg>Cys). Like flies — when the KID mutant is heterozygous with a WT receptor allele — young heterozygote KID mice have normal growth and glucose metabolism, yet they variably modify several hormones that are associated with slow aging in rodents and humans (Table 1).

### Modified hormones of murine KID mutants.

The murine InsR^R1109C^ and IGF-1R^R1096C^ KID substitutions alter several hormones associated with delayed aging in established mouse longevity models and in long-lived humans.

Heterozygote IGF1R^Arg1096Cys^ males have reduced plasma IGF1. In mammals, growth hormone produced by the anterior pituitary regulates the biosynthesis and release of IGF-1 by the liver and peripheral tissue to control mammalian growth. Several models of dwarf mice (Prop1, Pit1, GHRHR, and GHR) are long lived and all produce less IGF-1 (47–50). Compensatory upregulation of IGF-1 levels has been observed in some models with a tissue-specific KO of IGF-1R (51–53), while heterozygous *Igf1* mutant mice have been reported to have reduced circulating IGF-1 (54–56). Our model is potentially more like the second case, as we have full-body knock-in mutant mice and we observe decreased plasma IGF-1 levels in heterozygous IGF-1R^R1096C^ male mice. The absence of altered plasma IGF-1 in heterozygous IGF-1R^R1096C^ female mice may arise because they transcriptionally upregulate *Igf1r* and *Igf1* mRNA.

Heterozygote InsR^R1109C^ males and females have elevated plasma adiponectin. Adiponectin null mice have reduced health span and lifespan, while transgenic mice with high circulating adiponectin improve health span and lifespan (57). A pan-adiponectin receptor agonist (AdipoRon) administered for 6 weeks improves muscle function in aged male mice (58). Human female centenarians have higher plasma adiponectin than BMI-matched younger females, and plasma adiponectin in centenarians is positively associated with metabolic health biomarkers (59).

Heterozygote InsR^R1109C^ males have elevated plasma FGF21. FGF21 is an atypical fibroblast growth factor secreted by the liver during fasting. FGF21 elicits diverse starvation responses that are lacking in protein restricted FGF21-null mice (60). Notably, methionine restriction (MetR) increases plasma FGF21 in mice and humans (61, 62). MetR and FGF21 overexpression via a transgene induce hepatic fatty acid oxidation and ketogenesis, increase insulin sensitivity, decrease circulating IGF-1, and block somatic growth. Both manipulations extend lifespan in male and female mice (63, 64). Despite these parallels, some benefits of dietary MetR persist even in mice deficient for *Fgf21* and *Adipoq* (65), suggesting that adiponectin and FGF21 upregulation may reflect a methionine restricted state and have prolongevity effects, but they are not downstream effectors of lifespan extension by MetR.

Heterozygote IGF-1R^Arg1096Cys^ males have reduced plasma GDF15, which is considered to act as a stress responsive cytokine (32–34). GDF15 is normally upregulated during human and murine aging (34), while it is low in young individuals except when they experience chronic or acute illness (34). Reduced GDF15 in IGF1R^+/Arg10965Cys^ males at 4 months relative to WT suggests the KID mutant mice are biologically younger than matched controls.

Overall, the murine KID mutations variously affect a range hormones that are associated with attenuated aging in long-lived mice and humans. Aspects of these profiles parallel observations seen with dietary MetR.

### Genetic background.

We have explored substitutions in the KID in 1 genetic background, C57BL/6J. The knock-in mutations were produced in C57BL/6J and backcrossed to WT C57BL/6J mice to minimize the effect of off-target mutations. Previous work with igf1r and insulin receptor mutants shows that outcomes can vary among strain backgrounds (C57BL/6, 129Sv, and DBA) (66). Among these backgrounds, C57BL/6J may provide a stringent test condition because loss of either receptor produces severe phenotypes with obesity and diabetes. It is a sensitive background to detect deleterious metabolic phenotypes of the InsR^R1109C^ or IGF-1R^R1096C^ knock-in mutations. Here we found these KID substitutions (as WT heterozygotes) are metabolically healthy (IGF-1R mice) or have moderate hyperinsulinemia (IR mice). Potential future work with these receptor mutants will include additional metabolic tests (insulin tolerance test and hyperinsulinemic-euglycemic clamp studies) and alternative backgrounds such as outbred HET3 (67, 68).

### Models of mutant IR and IGFR to study aging.

Manipulations of insulin/IGF receptors typically used to extend lifespan in *C*. *elegans* and *Drosophila* also reduce insulin sensitivity, impede development (dauer, maturation, growth), and impair fecundity. We subsequently described a potentially novel mutant *Drosophila* receptor that slowed aging without such negative effects, and here sought to test similar alleles in mammals, being aware of how fly and mammalian insulin/IGF systems differ (69). Our work with a functional allomorph allele in mice contrasts with previous mouse studies that tested IR and IGF-1R loss-of-function genotypes. One early report found *igf1r*^+/–^ males and females were long-lived, but this benefit was not replicated in a different genetic background (9). KO of the IR in adipose tissue with the aP2 Cre driver extended mouse longevity, but the fat in these mice was developmentally abnormal (17, 18). Later studies demonstrated the aP2 promoter was promiscuous, and further studies using other fat-specific Cre drivers found detrimental effects of insulin receptor KO on metabolic health that accompanied opposite effects on lifespan (70, 71). Two teams mutated the insulin receptor substates IRS1 or IRS2 (72–74), but the gene attributed to extend lifespan differed between the labs. Mao et al. injected IGF-1R antibody into 18-mo old mice, which improved life expectancy and health span in females but not in males (23). Ambiguity also extends to humans where individuals with Laron syndrome, a human dwarf disease associated with IGF-1 deficiency, seem protected against cancer but otherwise appear to have normal lifespan (75).

Past studies of insulin and IGF receptor modulation of mouse aging have reduced the abundance of ligand, receptor, or receptor substrate, but these trails did not consistently slow aging. We propose an alternative approach to manipulate the quality of IR or IGF-1R signaling rather than their quantity. We show that heterodimer receptors composed of a mutant and WT protomer retain the ability to be autophosphorylated, although to a lesser extent. Nonetheless, ligand is equally able to induce pAkt in both IR and IGF-1R heterodimer containing cells, while the IGF-1R mutant impairs the induction of pErk. The IGF-1R KID may have a selective effect on MAPK signaling that cannot be revealed by fully knocking out an allele. The KID mutations may recapitulate targeting of the insulin/IGF/mTORC1 axis with rapamycin and the Ras/MEK/ERK axis with trametinib, where simultaneous inhibition of both axes additively extends lifespan in HET3 mice (76).

### Biological age of IGF1R KID mutant.

Progressive change in DNA methylation levels are sometimes used to systematically predict age and evaluate longevity interventions across different species, including humans (77, 78). Although epigenetic (or methylation) clocks are a powerful approach to estimate aging using blood or other tissues, they provide limited mechanistic insight. In contrast, recently developed transcriptome-based clocks can predict expected mortality and identify molecular pathways associated with the observed differences in biological age (40). These transcriptome-based clocks were trained on multiple interventions with known effects on lifespan derived from the Intervention Testing Program and other large survival studies. This approach not only predicts effects on mortality using gene expression data, but it also compares these changes to transcriptome profiles induced by lifespan-extending interventions. When we compared the transcriptional profiles from the livers of female IGF-1R^R1096C^ mice to characteristic signatures observed in established murine models of delayed aging, we found a positive correlation with the signature characteristic of calorie restriction and a negative association with signatures characteristic of aging-related degeneration (kidney aging and multitissue rodent aging). The correlation with the calorie restriction signature is not surprising, given the critical role of the insulin/IGF/mTORC1 axis in mediating the beneficial effects of calorie restriction (79). Interestingly, while kidney pathology is a hallmark manifestation of diabetes and insulin resistance, we observe that the IGF-1R KID mutant opposes kidney aging, consistent with its prolongevity effect and absence of detrimental effects on metabolic health.

To connect the transcriptional changes to specific processes that drive the aging process, the clocks were also trained on coregulated transcriptomic modules enriched for specific cellular pathways associated with aging and mortality (40). Applying module-specific transcriptomic mortality clocks to the IGF1R KID samples, we found that IGF-1R^R1096C^ females exhibited a statistically significant reduction in tAge in liver tissue associated with 39% of the module clocks. The most affected modules with the strongest tAge decrease included those related to lipid metabolism, VEGF signaling, mRNA splicing, mitochondria, NRF2 signaling, adaptive immunity, amino acid metabolism, heat stress response, and translation. Although we do not yet know how the IGF-1R KID mutation drives transcriptional changes in these modules, our recent work in *Drosophila* identified methionine metabolism as a critical downstream effector of the KID mutation (80), and methionine metabolism is known to regulate most of the processes identified in these transcriptionally altered modules (81). It should also be noted that data from transcriptome-based clocks are predictive of delayed aging but are not a substitute for an actual lifespan experiment, as some of the effects (both beneficial and detrimental) of the IGF-1R KID mutation may only appear later in life.

We suggest that understanding how insulin or IGF signaling can modulate mammalian aging may be advanced through analysis of a single amino acid substitution in the KID, as we demonstrated in *Drosophila* (5). Here we describe how a homologous substitution in the murine KID produces viable animals with normal growth and robust carbohydrate metabolism. These animals have encouraging early signatures of slowed aging, which may be fully revealed through further analysis across the lifespan of aging cohorts.

## Methods

*Sex as a biological variable*. Both male and female mice were used in this study.

*Igf1r-R1096C (AGG >TGC) and Insr-R1109C (AGG>TGC) mouse model generation*. C57Bl/6J mice (stock no. 000664) were purchased from Jackson Laboratory and housed with ad libitum diet and drinking water in a barrier facility (biosafety level 2) on 12-hour light-dark cycles. The protocol for generating genetically modified mice through CRISPR-Cas9 genome editing was approved by the IACUC of Brown University. All procedures involving mice were conducted in accordance with protocols approved by the IACUC of the University of Pittsburgh.

Three- to 4-week-old WT C57Bl/6J females were superovulated with pregnant mare serum gonadotropin (PMSG, Prospec Bio; I.P. 5IU/mouse) and human chorionic gonadotropin (HCG, Sigma-Aldrich; I.P. 5IU/mouse), followed by mating with WT stud males. At about 43.5 hours post-HCG administration, 2-cell embryos were harvested from successfully mated females and cultured in KSOM^AA^ medium (CytoSpring) before microinjection. The targeted mutation in mouse embryos was generated by cytoplasmic microinjection of both blastomeres with the mixed CRISPR-Cas9 reagents. The microinjection reagents (final concentration) were prepared in UltraPure DNase/RNase-Free distilled water (Invitrogen) following the steps of annealing crRNA and tracrRNA (molar ratio 1:1) according to IDT protocol, incubating Alt-R S.p. HiFi Cas9 Nuclease V3 (100 ng/μL, IDT) and annealed crRNA-tracrRNA (150 ng/μL, IDT) at room temperature for 10 minutes to form Cas9 ribonucleoprotein; and adding Ultramer DNA oligo template containing the mutation *Igf1r*-R1096C (AGG >TGC) or *Insr*-R1109C (AGG>TGC) (250 ng/μL, IDT). After microinjection, 25–30 embryos per recipient were transferred into CD1 pseudo-pregnant females.

The mice born from the microinjected embryos were genotyped to identify the targeted mutation. Genotypes were determined by Sanger sequencing analysis of the purified PCR products specifically amplified by a pair of primers with at least 150 nucleotides upstream and downstream of the targeted mutation site. The mice carrying the targeted mutation (founder mice, F0) were saved for germline transmission. Natural mating or in vitro fertilization (male founder mice only) of the founders with WT mice was performed to produce the F1 generation of mice. The heterozygous mice with the germline-transmitted targeted mutation were identified by genotyping. A heterozygous mouse was further backcrossed to WT C57Bl/6J mice to establish its colony. Supplemental Table 4 contains reagent sequence information.

*InsR^R1109C^ and IGF-1R^R1096C^ breeding*. To minimize the risk of off-target effects caused by CRISPR-Cas9, founder mice for both knock-in mutations were backcrossed with C57BL/6J WT mice (JAX, stock no. 000664) for 2 generations and selected for the presence of InsR^R1109C^ and IGF-1R^R1096C^ allele.

*Genotyping*. Tail tips were collected from pups, and DNA was extracted with the Qiagen DNeasy Blood & Tissue kit (no. 69506). PCR reactions were performed using Hot Start Taq Blue master mix (Apex Bioreserch), with forward primers *GGCAAGTGAGATTTGCTTGGG* for InsR^R1109C^ and *GGGTAGTTTCCCCGTTGCA*T for IGF-1R^R1096C^, and reverse primers *ACAGGGGTTGCAATTAGCACT* for InsR^R1109C^ and *CCATGACACGTGGTAGAGCA* for IGF-1R^R1096C^ with the following program: first cycle of denaturation at 95°C for 15 minutes and 28 cycles of denaturation at 95°C for 30 seconds, annealing at 59°C for 30 seconds, extension at 72°C for 1 minute, and a final extension at 72°C for 5 minutes. Fragments were visualized on 1% agarose gel using SYBER Safe gel stain (Thermo Fisher Scientific, S33102): InsR^R1109C^ amplicon of 476 bp and IGF-1R^R1096C^ amplicon of 588 bp. PCR products were sent for sanger sequencing (Azenta Genewiz) and point mutations were assessed with SnapGene 7.2 software. Simultaneous peaks for A and T and for G and C indicated individuals heterozygous for a WT codon AGG and a mutant codon tGC.

*Blood biochemical tests*. ELISA measured FGF-21 (EZRMFGF21-26K) (Millipore), adiponectin (ADIPOQ) (MRP300) (R&D Systems), insulin-like growth factor 1 (IGF-1) (MG100), leptin (MOB00B), and GDF15 (R&D Systems, DY6385). A colorimetric assay determined plasma triglycerides (TG) (TR22421) (Thermo Fisher Scientific) and total cholesterol (TR13421) (Thermo Fisher Scientific).

*IR/IGF-1R double knockout 293FT cells*. IR and IGF-1R double KO 293FT cells — generated in ref. 82 — were cultured in high-glucose (4.5 g/L) DMEM supplemented with 10% (v/v) FBS, 2 mM L-glutamine, and 1% penicillin/streptomycin. Cells were maintained at 37°C with a humidified atmosphere of 5% CO_2_.

*Cell-based receptor activation assays*. IR and IGF-1R activation assays were performed as previously described with some modifications (82, 83). For IR mutant generation, we used the short isoform of human IR (hIR-A) in pCS2-Myc vector resistant to IR gRNAs. IR R1109C mutation was generated by Q5 site-directed mutagenesis (pCS2-hIR-A-Myc, primer sequences: 5′- CCGTTCTCTGtgcCCAGAGGCTG -3′ and 5′- AGGTAGCTCTTCAGGTCTC -3′. For IGF-1R R1096C mutant generation, we used human IGF-1R in pCS2-Myc vector resistant to IGF1R gRNAs. IGF-1R R1096C mutant was generated by Q5 site-directed mutagenesis (pCS2-hIGF-1R-Myc, primer sequences: 5′- CCGGTCTCTGtgcCCAGAAATGG -3′ and 5′- AGATAACTTTTGAGATCGC -3′).

Plasmid transfection in double-knockout 293FT cells was performed with Lipofectamine 2000 (Invitrogen). After 1 day, the cells were serum-starved for 16–18 hours. Serum-starved cells were treated with the indicated concentrations of human insulin (Sigma, I2643) or human IGF-1 (PeproTech, 100-11).

After treatment, cells were incubated with cell lysis buffer [50 mM Hepes pH 7.4, 150 mM NaCl, 10% (v/v) Glycerol, 1% (v/v) Triton X-100, 1 mM EDTA, 10 mM sodium fluoride, 2 mM sodium orthovanadate, 10 mM sodium pyrophosphate, 0.5 mM dithiothreitol (DTT), 2 mM phenylmethylsulfonyl fluoride (PMSF)] supplemented with cOmplete Protease Inhibitor Cocktail (Roche) and PhosSTOP (Roche) on ice for 1 hour. Cell were centrifuged at 18,213*g* at 4°C for 20 minutes, and cell lysates analyzed by SDS-PAGE and Western blotting. Primary antibodies included anti-IR-pY1150/1151 (1:2000; 19H7, Cell Signaling, 3024), anti-IR (1:500; CT-3, Santa Cruz, sc-57342), anti-IGF-1R (1:1000; ZI001, Invitrogen, 39-6700), anti-AKT (1:2000; 40D4, Cell Signaling, 2920), anti-pS473 AKT (1:2000; D9E, Cell Signaling, 4060), anti-ERK1/2 (1:2000; L34F12, Cell Signaling, 4696), and anti-pERK1/2 (1:2000; 197G2, Cell Signaling, 4377). Secondary antibodies for quantitative Western blots included anti-rabbit immunoglobulin G (IgG) (H + L) (Dylight 800 conjugates, Cell Signaling, 5151) and anti-mouse IgG (H + L) (Dylight 680 conjugates, Cell Signaling, 5470). The membranes were scanned with the Odyssey Infrared Imaging System (LI-COR, Lincoln, NE). Levels of receptor autophosphorylation, pERK, and pAKT were normalized to total receptor, ERK, and AKT levels, respectively. For IR activation assay, intensities were shown relative to that of IR-WT treated with 50 nM insulin. For IGF-1R activation assay, intensities were shown relative to that of IGF-1R-WT treated with 50 nM IGF-1.

*RNA-Seq*. Reads were mapped to mouse genome (GRCm39) with STAR (version 2.7.11b) and counted via featureCounts (version 2.0.6). To filter out nonexpressed genes, we required at least 10 reads in at least 20% of samples separately for liver and skeletal muscle data. Differentially expressed genes were identified separately for each tissue and sex using 1-way ANOVA model through edgeR package (84). *P* values were adjusted for multiple testing with the Benjamini-Hochberg method.

*Transcriptomic signature analysis*. We conducted functional enrichment analysis to describe how the transcriptome changes in IGF-1R^R1096C^ heterozygous mice in various tissues and across sex, and we related these to established molecular signatures of aging, mortality, and lifespan regulation. We applied reference signatures from tissue-specific aging biomarkers of liver, kidney, and brain, and from multitissue biomarkers of chronological age and expected mortality, adjusted for chronological age (40). Additionally, we included hepatic signatures of expected maximum lifespan in rodents and signatures of individual longevity interventions, such as caloric restriction, genetic models of growth hormone deficiency, and rapamycin (41). For each tissue (liver and skeletal muscle) and sex, we ranked genes using a signed log-transformed *P* value metric estimated through differential expression analysis: –log(pv) × sgn(lfc), where pv and lfc are *P* value and logFC of a certain gene, respectively, and sgn is the signum function (equal to 1, –1, and 0 if value is positive, negative, or equal to 0, respectively). Ranked gene lists were subjected to GSEA using the fgsea package in R, with 10,000 permutations and multilevel Monte Carlo sampling. Gene sets were drawn from the HALLMARK, KEGG, and REACTOME collections of the Molecular Signatures Database (MSigDB). The same enrichment pipeline was applied to reference signatures of aging, mortality, and longevity interventions. Individual *P* values were adjusted for multiple testing with the Benjamini-Hochberg method. We computed Spearman correlations between NES to quantify similarities between signatures of IGF-1R^R1096C^ heterozygous mice and reference gene expression biomarkers.

### tAge analysis.

The filtered RNA-Seq data were processed with Relative Log Expression (RLE) normalization, log-transformation, and YuGene normalization (85). Missing expression values for clock genes not detected in the dataset were imputed using their corresponding precomputed average values. Normalized gene expression profiles were then centered to the median profile of control samples within each tissue. tAge for each sample was estimated using Elastic Net-based multitissue transcriptomic clocks of expected mortality (40). Module-specific transcriptomic clocks of expected mortality were applied to the scaled, relative gene expression profiles using the same framework. The resulting tAge values from module-specific clocks were standardized within each tissue and sex. One-way ANOVA was used to compare tAge estimates from composite and module-specific clocks. One-way ANOVA model including tissue as a covariate was used to compare composite clock tAges across tissues in females. Resulting *P* values were adjusted for multiple comparisons using the Benjamini-Hochberg method.

### Statistics.

For all comparisons between WT and heterozygous mice, data were assessed for normality (Shapiro-Wilk test) and evaluated by an unpaired 2-tailed Student’s *t* test. Welch’s correction was used if variances were not equal among groups. A Mann-Whitney *U* test was used when data were not normally distributed. Two-way ANOVA was used to evaluate the main and interaction effects upon phosphorylation events when ligands stimulated mutant receptors in cell culture. Metabolic cage data were normalized to weight and analyzed using 2-way ANOVA to compare across time cycles and genotypes. Alternatively (depending on whether all assumptions were satisfied: normality, homoscedasticity, and linearity), the data were also analyzed with ANCOVA, GAM, or GLM using lean mass as a covariate. Results from both analyses were nonsignificant; therefore, graphs based on the 2-way ANOVA were shown as a reference. All data are represented either by mean ± SD for normally distributed data or as median ± 95% CI for nonnormally distributed data (GraphPad Prism, version 10.4). Outliers were identified by the ROUT method. Growth curves were generated using a logistic 3-parameter model, and statistical differences between curves were determined using an equivalence test with JMP Pro software, version 17 (SAS Institute). ANCOVA was performed in R (version 4.5.0). The packages rstatix, mgcv, car, and emmeans were used for modeling, assumption testing, and post hoc comparisons.

All the key resources used in the study are listed in the Supplemental Table 5.

Extended meterials and methods can be found in the Supplemental Table 6.

### Study approval.

The protocol for generating genetically modified mice through CRISPR-Cas9 genome editing was approved by the IACUC of Brown University. Animal experiments were approved by and in compliance with the University of Pittsburgh IACUC.

### Data availability.

All data are available within the article and Supporting Data Values file, or available from the authors upon a request. RNA-Seq data reported in this work are available at NCBI GEO data repository under accession no. GSE303415.

## Author contributions

Conceptualization was contributed by MT and AAP. Methodology was contributed by AT, AM, JL, JKA, SJSR, GPA, EC, VNG, MJJ, MT, and AAP. Investigation was contributed by UHA, JKK, JLF, AT, AM, CH, SG, YG, IJS, JK, and DC. Visualization was contributed by UHA, AT, and EC. Supervision was contributed by MT and AAP. Writing of the original draft was contributed by UHA, MT, and AAP. Review and editing were contributed by UHA, AT, AM, SJSR, EC, VNG, MT, and AAP.

## Funding support

This work is the result of NIH funding, in whole or in part, and is subject to the NIH Public Access Policy. Through acceptance of this federal funding, the NIH has been given a right to make the work publicly available in PubMed Central.

- NIGMS R35 GM146869 (AAP)
- NIA R01 AG082801 (AAP and MT)
- NIA R03 AG075651 (AAP)
- R03 CA286521 (AAP)
- NIA P30 AG024827 pilot grant (AAP)
- Richard King Mellon Foundation award (AAP)
- NAM Healthy Longevity Catalyst Award (AAP)
- NIA R01 AG059563 (MT)
- NIA R01 AG069639 (MT)
- NIGMS R35 GM142937 (EC)
- Irma T. Hirschl Research Award (EC)
- Orentreich Foundation for the Advancement of Science Inc. and GPA18 (GA)
- Secretaría de Eduacón, Ciencia, Tecnología en Innovación de la Ciudad de México (SECTEI) (UHA)
- VNG is supported by NIA funding
- NIA R01 AG082696 after NIGMS R35 GM146869 (AAP)

## Supplementary Material

Supplemental data

Unedited blot and gel images

Supplemental table 1

Supplemental table 2

Supporting data values

## Figures and Tables

**Table 1:**
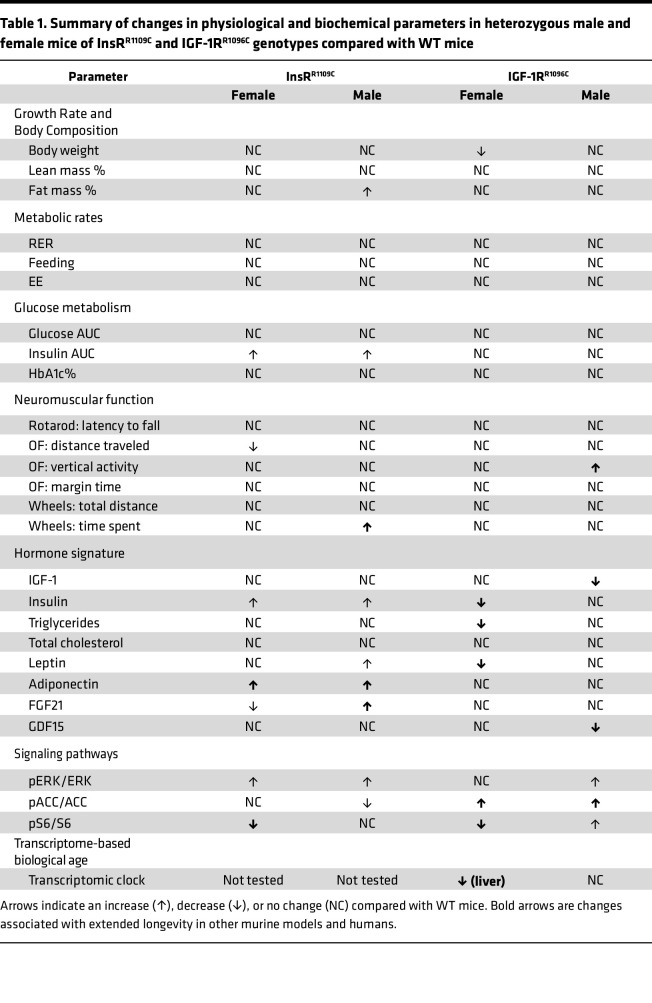
Summary of changes in physiological and biochemical parameters in heterozygous male and female mice of InsR^R1109C^ and IGF-1R^R1096C^ genotypes compared with WT mice

## Version 1 11/11/2025

In-Press Preview

## Version 2 12/22/2025

Electronic publication
