# Supplementary material for: Mutation in IR or IGF1R produces features of long-lived mice while maintaining metabolic health
Source: JCI Insight. 2025 Nov 11;10(24):e189683. doi: 10.1172/jci.insight.189683 (PMC12890504; doi:10.1172/jci.insight.189683)
Supplement: Supplemental data [file jciinsight-10-189683-s219.pdf]

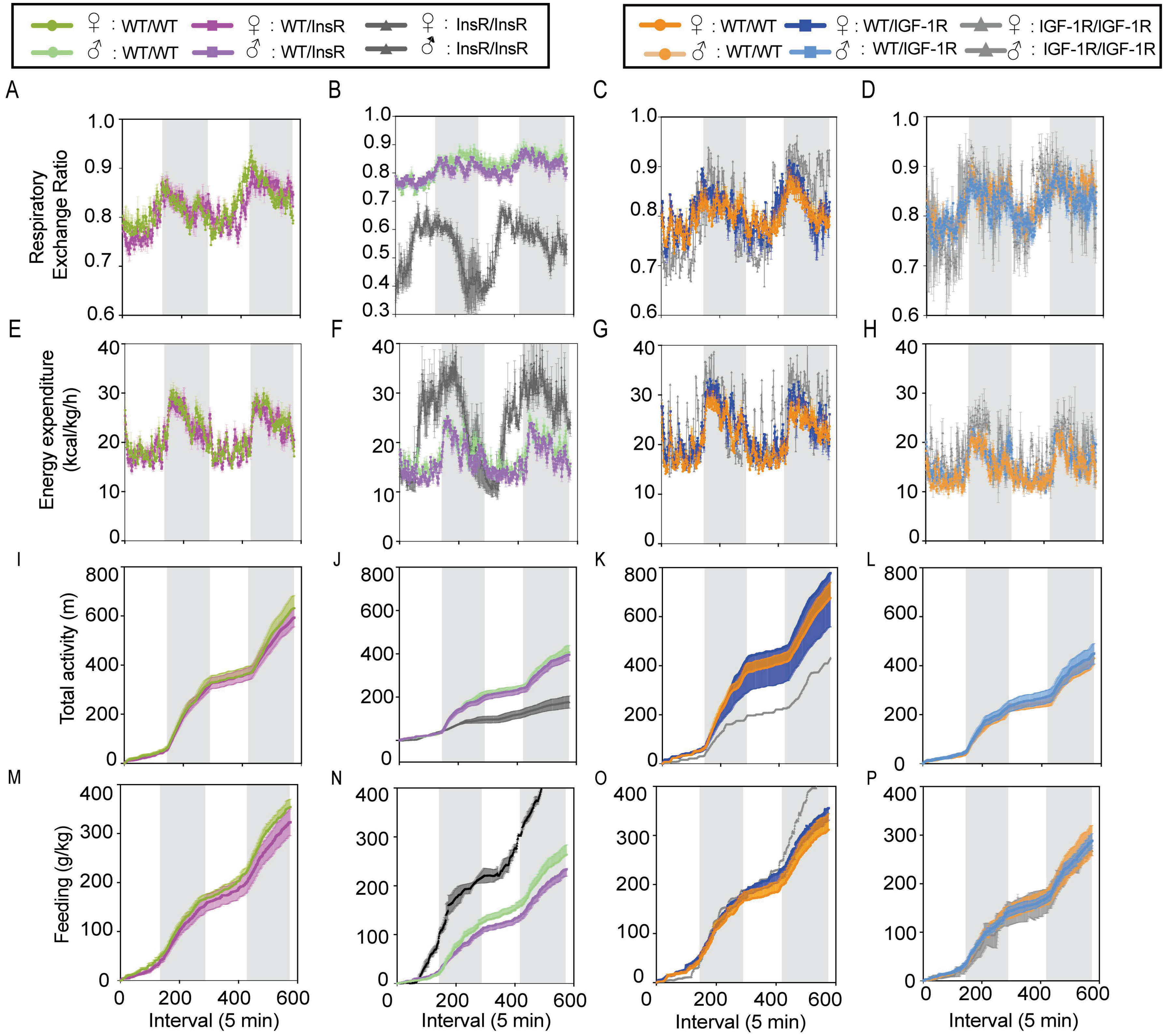

♀ : WT/WT
♂ : WT/WT
♀ : WT/InsR
♂ : WT/InsR
♀ : InsR/InsR
♂ : InsR/InsR
♀ : WT/WT
♂ : WT/WT
♀ : WT/IGF-1R
♂ : WT/IGF-1R
♀ : IGF-1R/IGF-1R
♂ : IGF-1R/IGF-1R
A
B
C
D
Respiratory Exchange Ratio
E
F
G
H
Energy expenditure (kcal/kg/h)
I
J
K
L
Total activity (m)
M
N
O
P
Feeding (g/kg)
Interval (5 min)

Supplementary Figure 1. $InsR^{R1109C}$ and $IGF\text{-}1R^{R1096C}$ heterozygous mice maintain normal metabolic rates.

Respiratory exchange ratio (RER) in 5 min intervals over 48 hr. **(A)** female and **(B)** male $InsR^{R1109C}$; **(C)** female and **(D)** male $IGF\text{-}1R^{R1096C}$. Energy expenditure (EE) in 5 min intervals over 48 hr (per kg of lean mass): **(E)** female and **(F)** male $InsR^{R1109C}$; **(G)** female and **(H)** male $IGF\text{-}1R^{R1096C}$. Total activity in 5 min intervals over 48 hr: **(I)** female and **(J)** male $InsR^{R1109C}$; **(K)** female and **(L)** male $IGF\text{-}1R^{R1096C}$. Feeding in 5 min intervals over 48 hr (per kg of lean mass): **(M)** female and **(N)** male $InsR^{R1109C}$; **(O)** female and **(P)** male $IGF\text{-}1R^{R1096C}$.

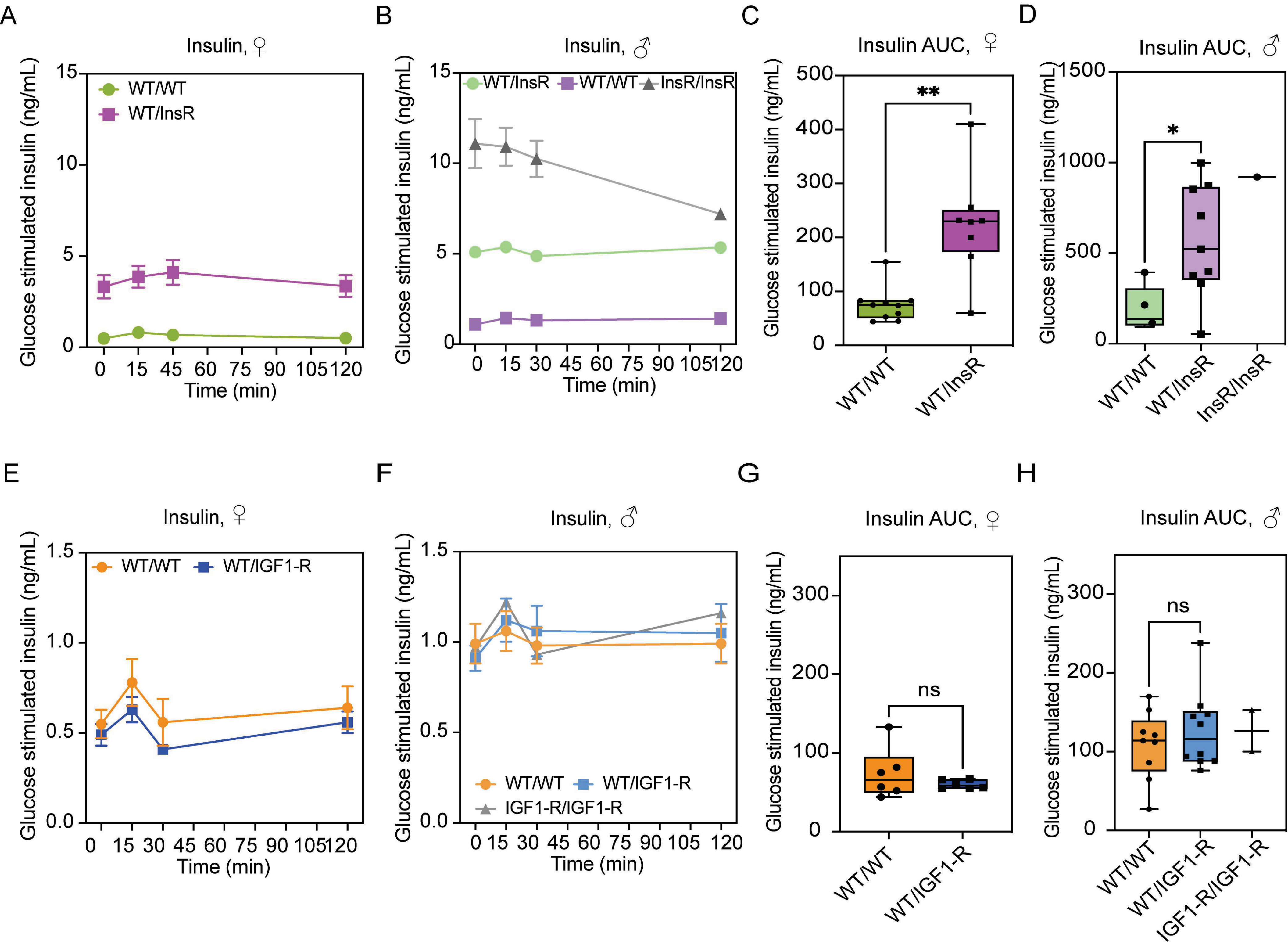

A
Insulin, ♀
WT/WT
WT/InsR
Glucose stimulated insulin (ng/mL)
Time (min)
B
Insulin, ♂
WT/InsR
WT/WT
InsR/InsR
Glucose stimulated insulin (ng/mL)
Time (min)
C
Insulin AUC, ♀
**
Glucose stimulated insulin (ng/mL)
WT/WT
WT/InsR
D
Insulin AUC, ♂
*
Glucose stimulated insulin (ng/mL)
WT/WT
WT/InsR
InsR/InsR
E
Insulin, ♀
WT/WT
WT/IGF1-R
Glucose stimulated insulin (ng/mL)
Time (min)
F
Insulin, ♂
WT/WT
WT/IGF1-R
IGF1-R/IGF1-R
Glucose stimulated insulin (ng/mL)
Time (min)
G
Insulin AUC, ♀
ns
Glucose stimulated insulin (ng/mL)
WT/WT
WT/IGF1-R
H
Insulin AUC, ♂
ns
Glucose stimulated insulin (ng/mL)
WT/WT
WT/IGF1-R
IGF1-R/IGF1-R

Supplementary Figure 2. InsR^{R1109C} and IGF-1R^{R1096C} heterozygous mice maintain normal glycated hemoglobin.

Glucose (1.5g/kg) stimulated insulin in female **(A)** and male **(B)** InsR^{R1109C}. Area under the curve (AUC) for glucose-stimulated insulin in female **(C)** and male **(D)** InsR^{R1109C}. Glucose (1.5g/kg) stimulated insulin in female **(E)** and male **(F)** IGF-1R^{R1096C}. Area under the curve (AUC) for glucose-stimulated insulin in female **(G)** male **(H)** IGF-1R^{R1096C}.

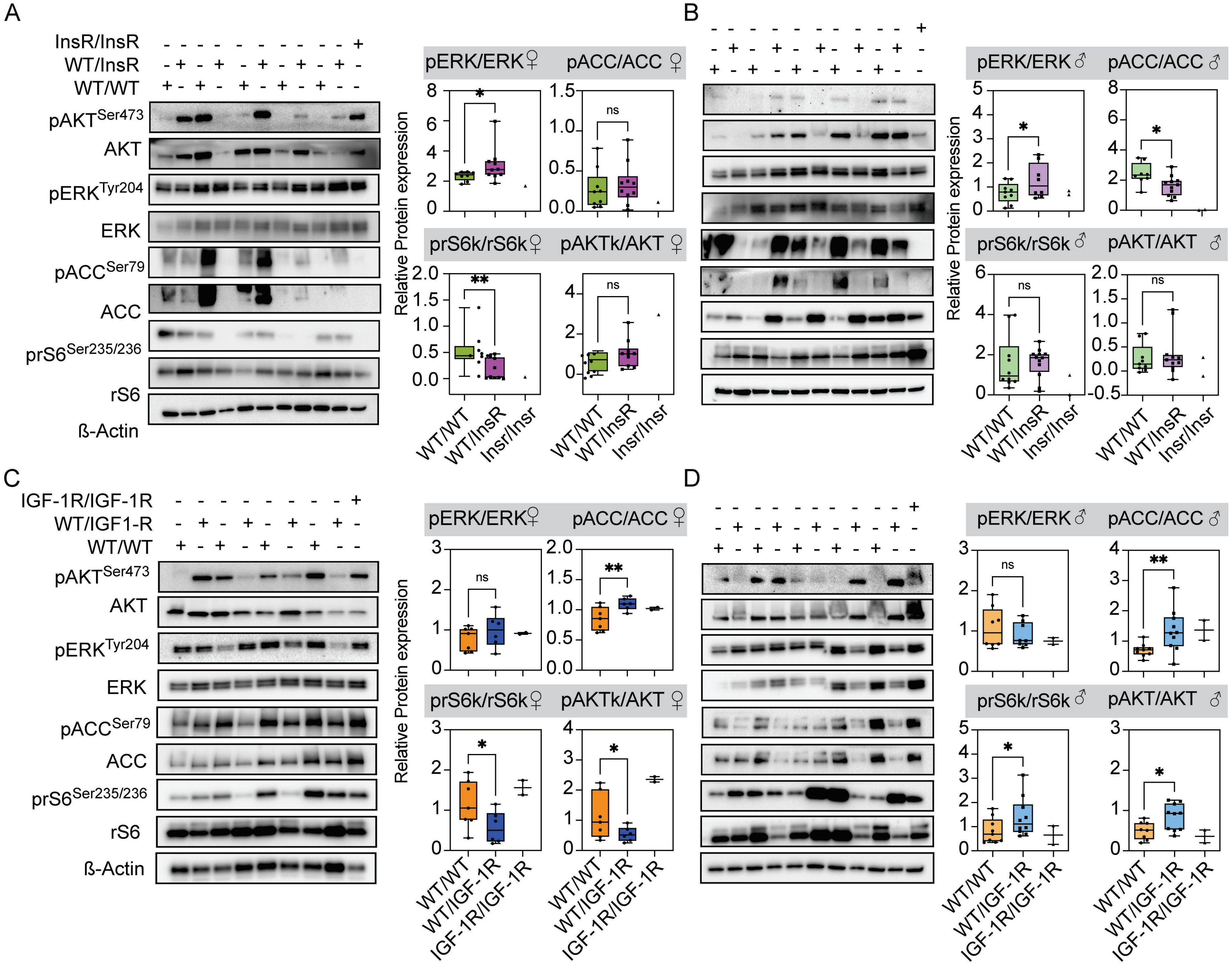
A
InsR/InsR
WT/InsR
WT/WT
pAKT^Ser473
AKT
pERK^Tyr204
ERK
pACC^Ser79
ACC
prS6^Ser235/236
rS6
ß-Actin
pERK/ERK♀
pACC/ACC ♀
prS6k/rS6k♀
pAKTk/AKT ♀
Relative Protein expression
ns
WT/WT
WT/InsR
Insr/Insr
B
pERK/ERK♂
pACC/ACC ♂
prS6k/rS6k♂
pAKT/AKT ♂
C
IGF-1R/IGF-1R
WT/IGF1-R
WT/WT
pAKT^Ser473
AKT
pERK^Tyr204
ERK
pACC^Ser79
ACC
prS6^Ser235/236
rS6
ß-Actin
pERK/ERK♀
pACC/ACC ♀
prS6k/rS6k♀
pAKTk/AKT ♀
WT/WT
WT/IGF-1R
IGF-1R/IGF-1R
D
pERK/ERK♂
pACC/ACC ♂
prS6k/rS6k♂
pAKT/AKT ♂

Supplementary Figure 3. Liver AMPK and mTORC1 activity in $InsR^{R1109C}$ and $IGF\text{-}1R^{R1096C}$ heterozygous mice.

Representative blot showing five wildtype, five heterozygotes and one homozygous mouse after 6 hr morning fast: $InsR^{R1109C}$ **(A)** Females, **(B)** Males; $IGF\text{-}1R^{R1096C}$ **(C)** Females and **(D)** Males. Relative $pERK^{Tyr204}$/ERK, $pACC^{Ser79}$/ACC, $PAKT^{Ser473}$/AKT and $prS6^{Ser235/236}$/rS6 respectively for $InsR^{R1109C}$ females and males; and $IGF\text{-}1R^{R1096C}$ females and males. Ratios of phosphorylated protein to total protein. Box plot for minimum and maximum with median. T-test sample size: Females WT/WT n= 9 and WT/InsR n= 10; Males WT/WT n= 8-10 and WT/InsR n =8-12; Females WT/WT n= 7 and WT/IGF-1R n= 6; Males WT/WT n= 10 and WT/IGF-1R n= 9-10). * $p < 0.05$; **, $p < 0.01$; ***, $p < 0.001$: ns, no significant differences.

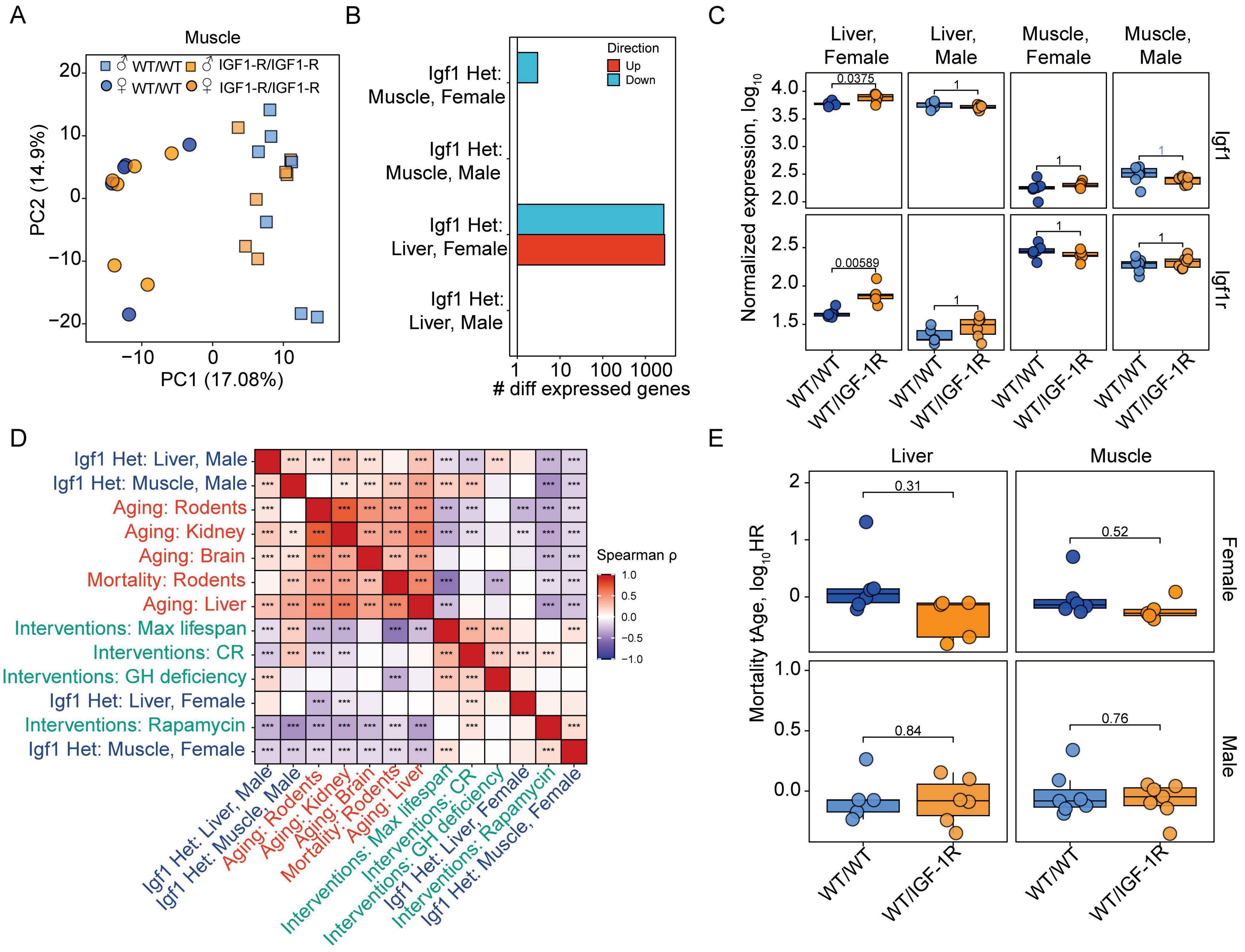

A
Muscle
♂ WT/WT
♂ IGF1-R/IGF1-R
♀ WT/WT
♀ IGF1-R/IGF1-R
PC2 (14.9%)
PC1 (17.08%)
20
10
0
−10
−20
−10
0
10
B
Direction
Up
Down
Igf1 Het: Muscle, Female
Igf1 Het: Muscle, Male
Igf1 Het: Liver, Female
Igf1 Het: Liver, Male
1
10
100
1000
diff expressed genes
C
Liver, Female
Liver, Male
Muscle, Female
Muscle, Male
Normalized expression, log10
Igf1
Igf1r
0.0375
0.00589
1
WT/WT
WT/IGF-1R
4.0
3.5
3.0
2.5
2.0
1.5
D
Igf1 Het: Liver, Male
Igf1 Het: Muscle, Male
Aging: Rodents
Aging: Kidney
Aging: Brain
Mortality: Rodents
Aging: Liver
Interventions: Max lifespan
Interventions: CR
Interventions: GH deficiency
Igf1 Het: Liver, Female
Interventions: Rapamycin
Igf1 Het: Muscle, Female
Spearman ρ
1.0
0.5
0.0
−0.5
−1.0
E
Liver
Muscle
Female
Male
Mortality tAge, log10HR
0.31
0.52
0.84
0.76
2
1
0
1.0
0.5
0.0
WT/WT
WT/IGF-1R

Supplementary Figure 4. Estimated biological age in heterozygous IGF-1R^{R1096C} females

(A) Principal component analysis (PCA) of skeletal muscle gene expression profiles from WT and IGF-1R^{R1096C} heterozygous mice. **(B)** Number of differentially expressed genes (Benjamini-Hochberg adjusted p-value < 0.05) between WT and IGF-1R^{R1096C} heterozygous mice identified by tissue and sex. **(C)** *Igf1* and *Igf1r* expression in muscle and liver of WT and IGF-1R^{R1096C} heterozygous mice. Differential expression assessed with ANOVA model using edgeR; p-values adjusted for multiple comparisons with Benjamini-Hochberg. **(D)** Spearman correlation of enriched pathways within organs of IGF-1R^{R1096C} heterozygous mice (blue) that are associated with signatures of aging, with mortality (red), and with lifespan-extending interventions (green). Pairwise Spearman correlations were calculated based on NES values determined for each signature via GSEA. * p.adjusted < 0.05, ** p.adjusted < 0.01, *** p.adjusted < 0.001. **(E)** Mortality transcriptomic age (tAge) of tissue from WT and IGF-1R^{R1096C} heterozygous female mice by organ and sex, assessed with the rodent multi-tissue Elastic Net (EN) clock. tAges between the groups were compared with ANOVA, and corresponding BH-adjusted p-values.

Supplementary Table 1. Differentially expressed genes in livers and muscles of heterozygous IGF-1R^{R1096C} mice.

Supplementary Table 2. Gene set enrichment analysis (GSEA) from IGF-1R^{R1096C} liver samples.

Supplementary Table 3. The corresponding numbers for mouse and human IR and IGF-1R with and without signal peptide.

Supplementary Table 4. Sequence details of reagents for *Igf1r* and *Insr* gene replacement.

Supplementary Table 5. KEY RESOURCES TABLE.

Supplementary Table 6. Extended Materials and Methods.

Supplementary Table 3. The corresponding numbers for mouse and human IR and IGF-1R with and without signal peptide.

Protein	With signal peptide (precursor)	Without signal peptide (mature)
Human IR-A	R1107	R1080
Human IR-B	R1119	R1092
Mouse IR-A	R1109	R1082
Mouse IR-B	R1121	R1094
Human IGF1R	R1095	R1065
Mouse IGF1R	R1096	R1066

Supplementary Table 4. Sequence details of reagents for *Igf1r* and *Insr* gene replacement.

**Mouse model**	***Igf1r*-R1096C (AGG >tGc)**
Alt-R™ CRISPR-Cas9 crRNA (PAM)	AAAGTTATCTCCGGTCTCTG (AGG)
Ultramer™ DNA oligos (reverse strand)	GCCAGCTCCTGAAAAAAGTGGACCAAGCAAGATAACACAGCGAGGAACAAAACTGACCTCCACTTCTGGgCaCAGAGACCGGAGATAACTTTTGAGATCACCGCGTGTCATTAGTTCCATGATGACCAGGGTGGGCTGGCCTTGGGATACCACACCCAG
Genotyping primer, forward	GGGTAGTTTCCCCGTTGCAT
Genotyping primer, reverse	CCATGACACGTGGTAGAGCA
**Mouse model**	***Insr*-R1109C (AGG>tGc)**
Alt-R™ CRISPR-Cas9 crRNA (PAM)	AAAGTCACCTCCGTTCTCTG (AGG)
Ultramer™ DNA oligos (reverse strand)	GCATTTAGTGAGGTTGGTATACGTAAGATCAGGTACCCTGTTATGGGTCTTACCTAGAGGCAGCTTACCTCAGCATCTGGgCaCAGAGAACGGAGGTGACTTTTCAGGTCTCCATGAGCCATCAATTCCATCACTACCAGCGTTGGCTGTCCTTTG
Genotyping primer, forward	GGCAAGTGAGATTTGCTTGGG
Genotyping primer, reverse	ACAGGGGTTGCAATTAGCACT

Supplementary Table 5. KEY RESOURCES TABLE

REAGENT or RESOURCE	SOURCE	IDENTIFIER
**Mouse strains**		
C57BL/6J wildtype (WT) mice	The Jackson Laboratory	#:**000664**
IGF1R-R1096C	In-house made, Brown Tg Core	NA
InsR-R1109C	In-house made, Brown Tg Core	NA
Oligo 22007-F1: GGCAAGTGAGATTTGCTTGGG	IDT	NA
Oligo 22007-R1: ACAGGGGTTGCAATTAGCACT	IDT	NA
22006-F1: GGGTAGTTTCCCCGTTGCAT	IDT	NA
22006-R1: CCATGACACGTGGTAGAGCA	IDT	NA
**Plasmids**		
pCS2-hInsR	(1)	NA
pCS2-hInsR-R1107C-Myc (labeled as R1109C)	This paper	NA
pCS2-hIGF1R	(2)	NA
pCS2-hIGF1R-R1095C (labeled as R1096C)	This paper	NA
hInsR R1109C-F	CCGTTCTCTGtgcCCAGAGGCTG	NA
hInsR R1109C-R	AGGTAGCTCTTCAGGTCTC	NA
hIGF1R-R1095C-F	CCGGTCTCTGtgcCCAGAAATGG	NA
hIGF1R-R1095C-R	AGATAACTTTTGAGATCGC	NA

**Hot Start Taq BLUE** Master Mix	**Apex Bioresearch**	Cat #: 42-144
Q5 site-directed mutagenesis	NEB	E0554S
SnapGene 7.2	From Dotmatics; available at snapgene.com)	University of Pittsburgh license
**DNeasy Blue & Tissue kit**	**QiaGen**	69506
RIPA Buffer (10X)	Cell signaling	9806
Pierce™ Protease Inhibitor Mini Tablets, EDTA-free	Thermo Fisher	A32955
PhosSTOP Easy pack	Roche	4906845001
SYBR™ Safe DNA Gel Stain	Thermo Fisher	S33102
BenchMark™ Pre-stained Protein Ladder	Thermo Fisher	0748010
Trans-Blot Turbo RTA Mini 0.2 µm PVDF Transfer Kit	Bio-RAD	1704272
ProSignal Femto	Prometheus	20302
Mouse Hemoglobin A1c kit	Crystal Chem	80310
Mini-Protean TGX gels	Bio-Rad	4561095
Zirconium Oxide Beads 1.0 mm	Next Advance	ZROB10
2x Laemmli Sample Buffer	Bio-rad	1610737EDU
Phospho-p44/42 MAPK (Erk1/2) (Thr202/Tyr204)	Cell Signaling	4370
p44/42 MAPK (Erk1/2)	Cell Signaling	4695

Phospho-Acetyl-CoA Carboxylase (Ser79)	Cell Signaling	3661
pY 1150/1151 IR (pY1135/1136 IGF1R)	Cell signaling	3024
IR (CT-3)	Santa Cruz	sc-57342
IGF1R (ZI001)	Invitrogen	39-6700
pERK1/2 (197G2)	Cell signaling	4377
ERK1/2 (L34F12)	Cell signaling	4696
pAKT (D9E)	Cell signaling	4060
AKT (40D4)	Cell signaling	2920
Acetyl-CoA Carboxylase	Cell Signaling	3676
Phospho-S6 Ribosomal Protein	Cell Signaling	2211
S6 Ribosomal Protein	Cell Signaling	2317
Sheep Anti-Mouse IgG, HRP	Prometheus Protein Biology Products	84-848
anti-rabbit IgG (H + L) (Dylight 800 conjugates)	Cell Signaling	5151
anti-mouse IgG (H + L) (Dylight 680 conjugates)	Cell Signaling	5470

1. Choi E, et al. Mitotic checkpoint regulators control insulin signaling and metabolic homeostasis. Cell. 2016;166(3):567–581.

2. Li J, et al. Structural basis of the activation of type 1 insulin-like growth factor receptor. Nat Commun. 2019;10(1):4567.

Supplementary Table 6. Extended Materials and Methods.

Growth and weight

Mice were weighed weekly to the nearest 0.1g from age 2 weeks up to 4 months of age. To calculate the growth rates of mice, we fitted a curve to the relationship between weight (g) and age (weeks) using a logistic 3 -parameter model. Differences among genotypes were determined by comparing the growth rate, inflection point, and asymptote of the curve using equivalence tests.

Body Composition

Body composition (percent fat mass and percent lean mass) was assessed once at 4 months of age by NMR (EchoMRI, Echo Medical System). Non-anesthetized mice were placed in a restraint cylinder and scanned for approximately 2 min. Data expressed as percent of body mass.

Metabolic cage analysis

The Sable Systems Promethion Multi-plexed Metabolic Cage system was used to evaluate feeding, activity, energy expenditure (EE), and respiratory exchange ratio (RER) by indirect calorimetry. Mice were individually housed during 72 h, with the first 24 h for acclimatation and the subsequent 48hrs data to calculate values across light and dark cycle for each metabolic parameter.

In vivo glucose homeostasis

Glucose tolerance tests were conducted in the morning following a 6 hr fast. Mice received an intraperitoneal injection of glucose at a dose of 1.5 g/kg, and blood samples were collected from the tail at specific time points to measure plasma insulin and glucose.

Rotarod

Mice were tested on a rotarod (Ugo-Basile, model 47600) started at 4 RPM and accelerated to 40 RPM over a 300-second period. Each mouse underwent three consecutive trials, with approximately a 1-minute interval between. Latency to fall was recorded. Mice that fell immediately upon placement, before the rod began to accelerate, were given a score of 0 seconds for that trial.

Open Field

Mice acclimated to the testing room were placed in a Versamax Open Field Arena (40 cm × 40 cm × 40 cm; Omnitech Electronics, OH, United States). Infrared beams tracked their movements, recording distance traveled (in cm), vertical activity, and time spent in the arena perimeter and center. Data were collected in 5-minute intervals over a total period of 60 minutes.

Home Cage Wheel Running

Subjects were individually housed with a wireless running wheel affixed with a revolution sensor (Lafayette Instruments, Actimetrics Wireless Low-profile running wheel) and left undisturbed for three consecutive nights except for daily welfare checks.

Data were analyzed using ClockLab Data Software (Lafayette Instruments) in 1 min epochs and calculated as the cumulative time spent running and the average total distance traveled over 30 min periods.

Homogenization and Protein Extraction for Western blotting

Mice were sacrificed in small groups over four consecutive days at the same time of day. Sacrifices were conducted within two hours. Liver tissue (20-30mg) was homogenized in RIPA protein extraction reagent (Cell signaling); 1 mL per 100 mg of tissue weight) supplemented with protease and phosphatase inhibitors cocktail (Sigma-Aldrich) by using Zirconium Oxide Beads (Next Advance). Total protein concentration was measured using bicinchoninic acid (BCA; Pierce). Lysates were aliquoted and stored at -80C.

For analysis, protein lysates were boiled with loading buffer (Laemmli buffer and ßME) in a 1:1 ratio for 10 min at 95ºC. Proteins (20µg or 100µg) were separated on 4-20% mini-Protean TGX gels (Bio-Rad) SDS-PAGE, transferred to PVDF membranes for Trans-Turbo transfer (Bio-Rad), and incubated with specific primary antibodies against anti-p44/42 MAPK (Erk1/2), anti-Phospho-p44/42 MAPK (Erk1/2) (Thr202/Tyr204, anti-phospho-ACC, anti-ACC, anti-Phospho-S6 ribosomal, anti-S6 Ribosomal, anti-phospho-Akt (Thr308), anti-Akt (all Cell signaling). Membranes were washed three times with PBS-Tween and incubated with anti-Mouse HRP secondary antibody (Prometheus Protein Biology Products) for 1 h. After three consecutive washes, the blots were developed using a commercial chemiluminescence reagent ProSignal Femto (Prometheus). The proportion of these proteins was quantified by densitometric analysis using ImageJ (1.51v).

RNA isolation

Total RNA from liver and gastrocnemius muscle tissues of female WT and IGF1R heterozygous mice was isolated with 1 mL TRIzol Reagent (Invitrogen, CA, USA) and PureLink RNA Mini Kit (Invitrogen, CA, USA). Phase separation was performed with chloroform, and the aqueous phase recovered with 70% ethanol. DNase treatment was performed directly on the column. Eluted RNA quality was within the 260/280 of 1.9 and 2.0.
