# Supplementary material for: Mutation in IR or IGF1R produces features of long-lived mice while maintaining metabolic health
Source: JCI Insight. 2025 Nov 11;10(24):e189683. doi: 10.1172/jci.insight.189683 (PMC12890504; doi:10.1172/jci.insight.189683)

Membranes for Figure 7a

pY IGF1R

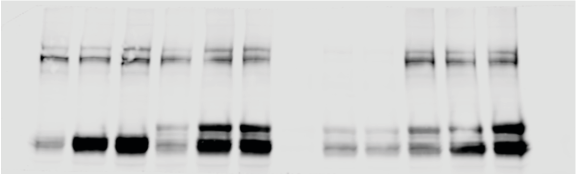

IGF1R

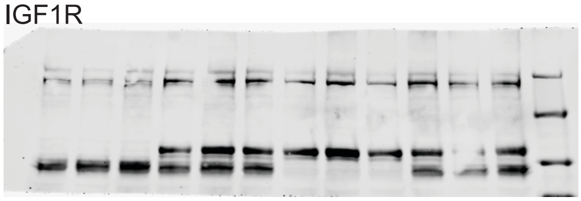

pAKT

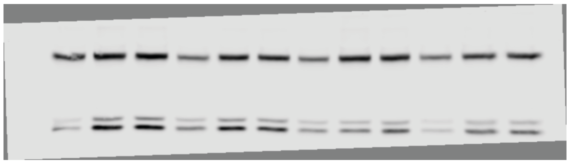

AKT

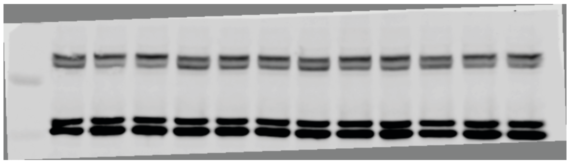

pERK

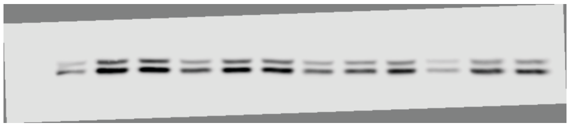

ERK

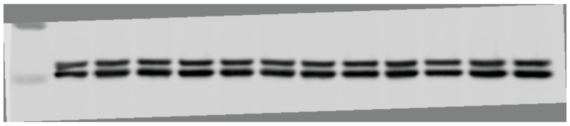

Membranes for Figure 7c

pY IR

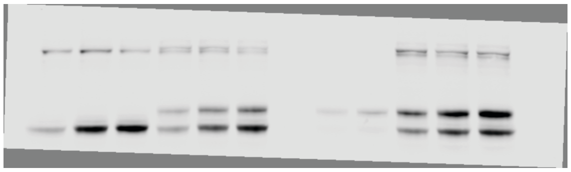

IR

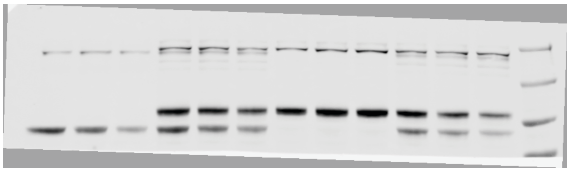

pAKT

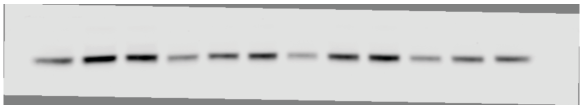

AKT

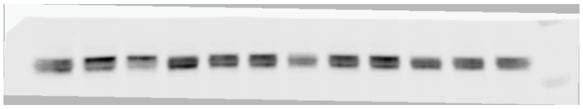

pERK

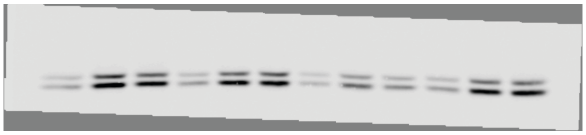

ERK

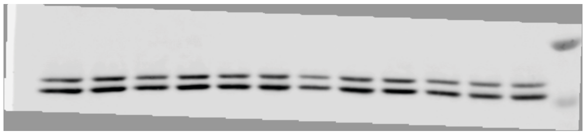

Membranes for Supplementary Figure 3a

pACC Ser 79

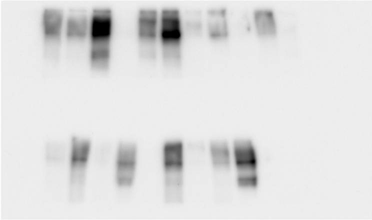

ACC

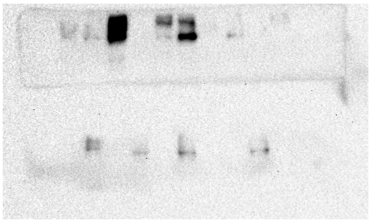

prS6 Ser235/236

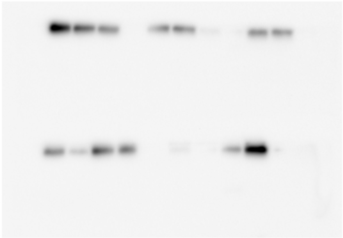

S6

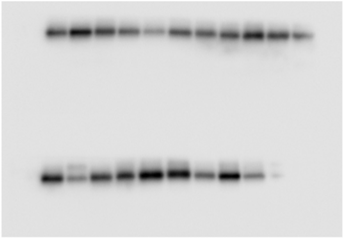

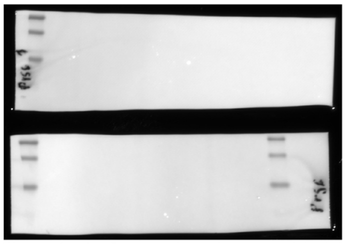

pAKT Ser473

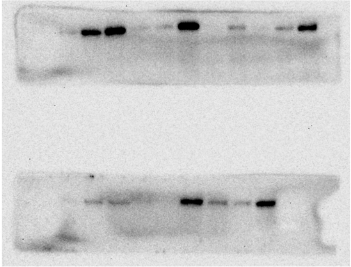

AKT

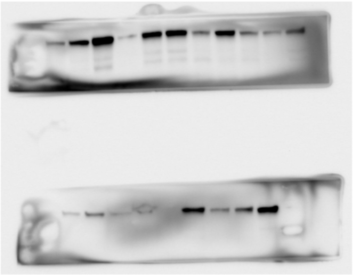

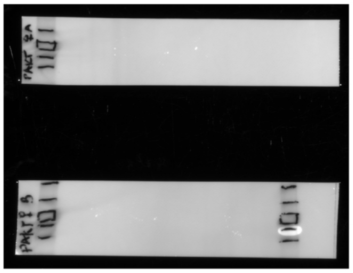

pERK Thr202/Tyr204

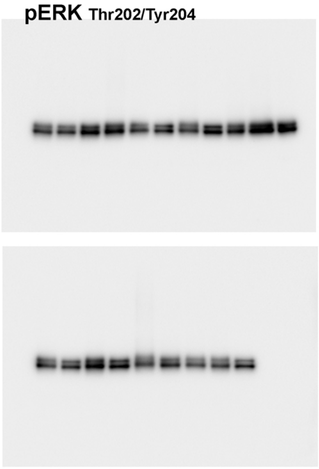

ERK

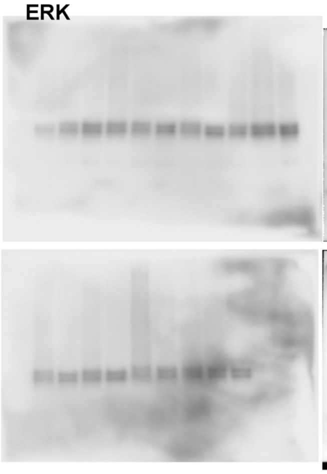

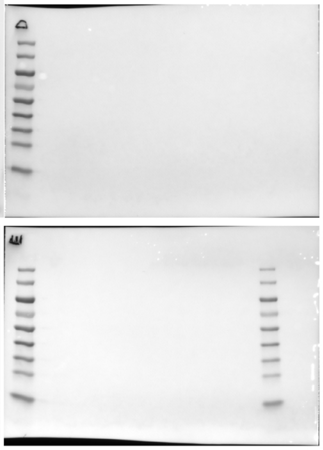

ß-Actin

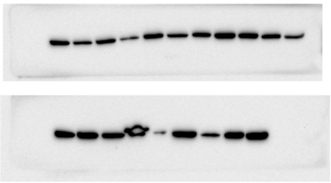

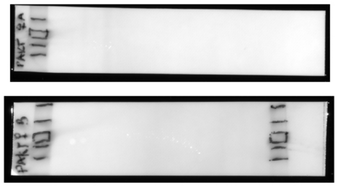

InsR_Males Membranes for Supplementary Figure 3b

pACC Ser79

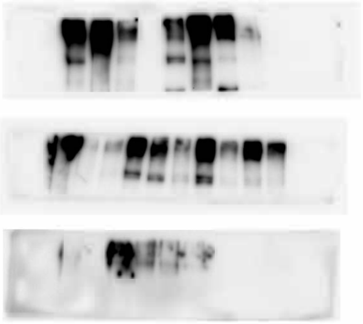

ACC

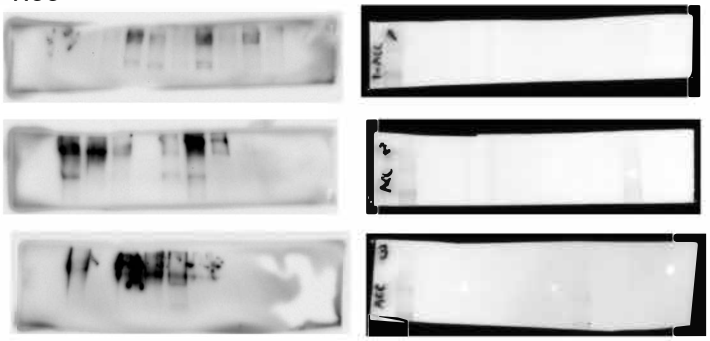

prS6 Ser235/236

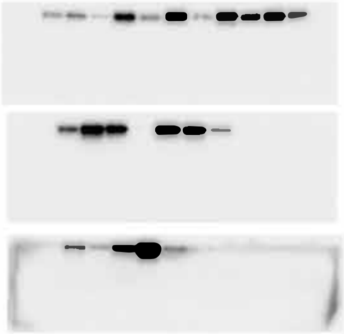

S6

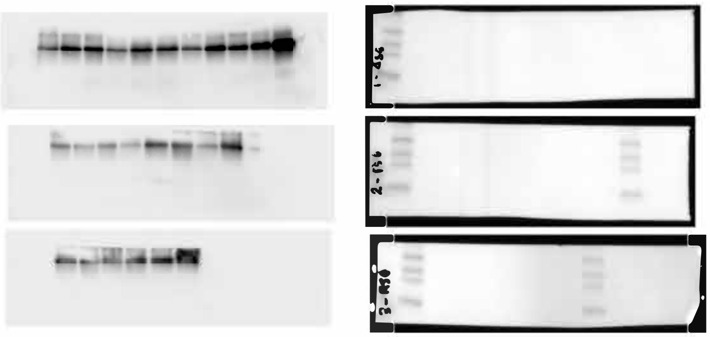

AKT

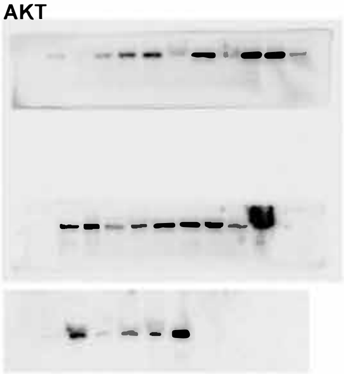

pAKT Ser473

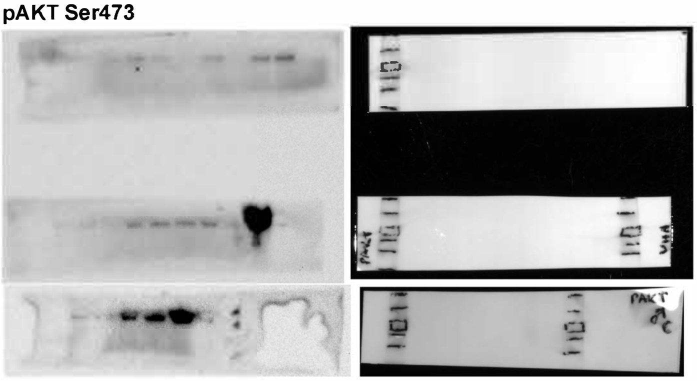

ERK

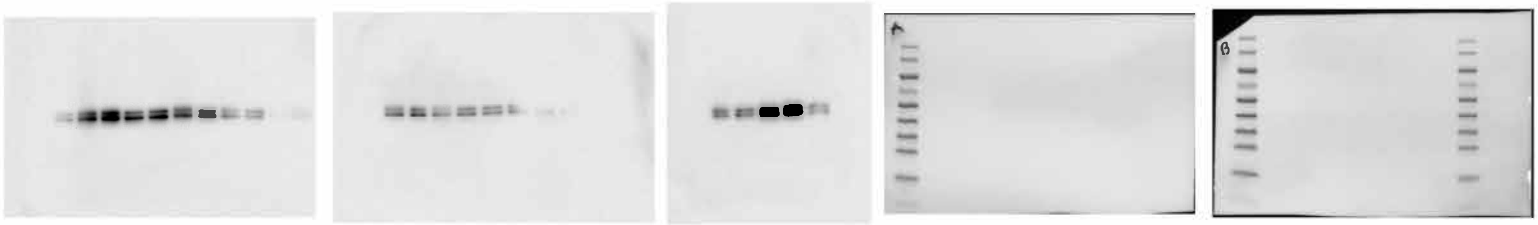

pERK Thr202/Tyr204

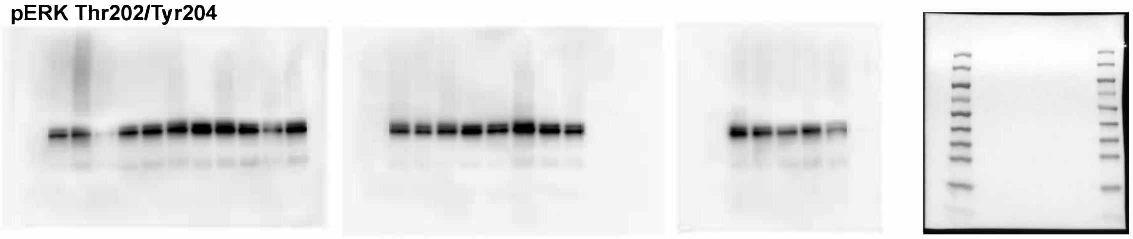

ß-Actin

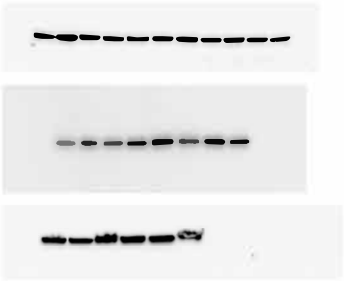

Igf1 Females Membranes for Supplementary Figure 3c

pERK Thr202/Tyr204

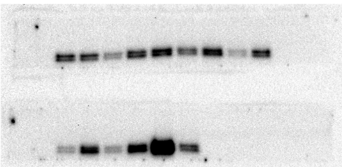

ERK

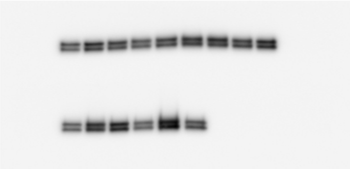

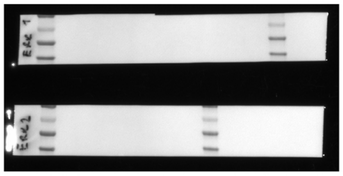

S6

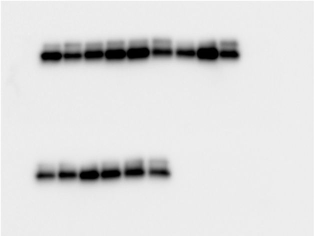

prS6 Ser235/236

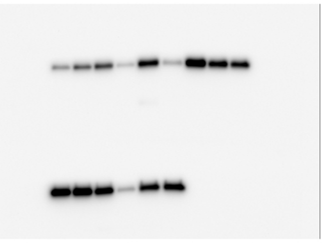

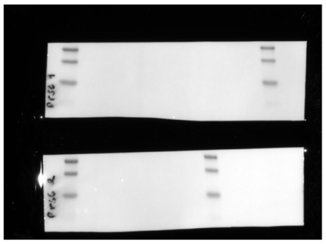

pACC Ser79

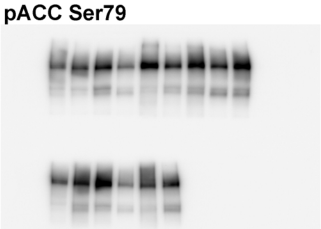

ACC

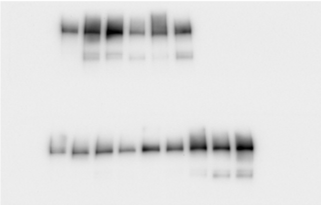

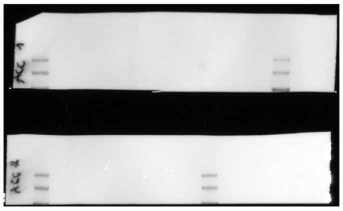

pAKT Ser473

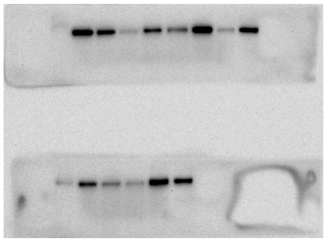

AKT

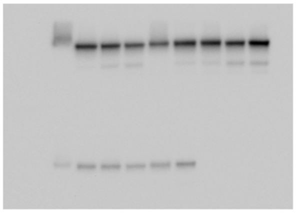

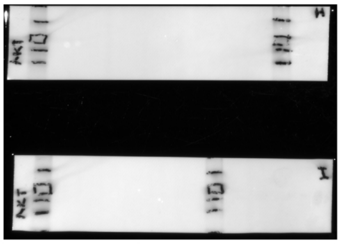

ß-Actin

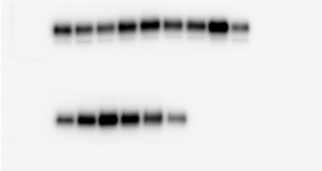

Igf1 Males Membranes for Supplementary Figure 3c

pERK Thr202/Tyr204

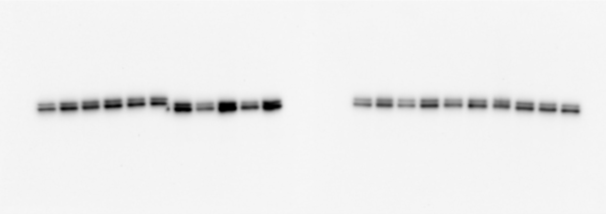

ERK

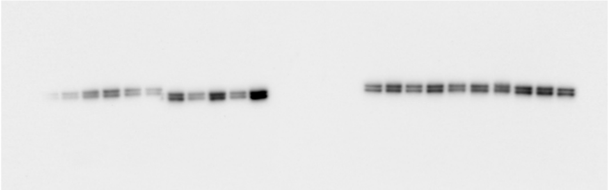

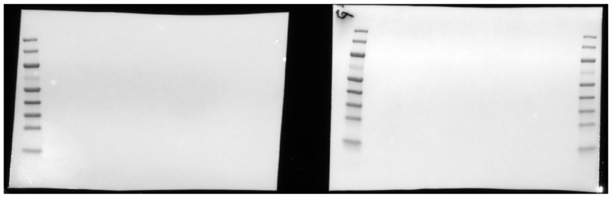

prS6 Ser235/236

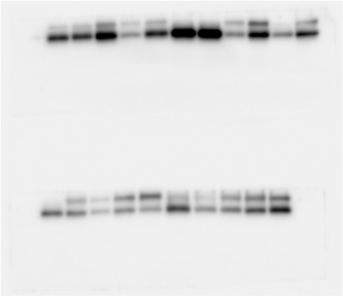

S6

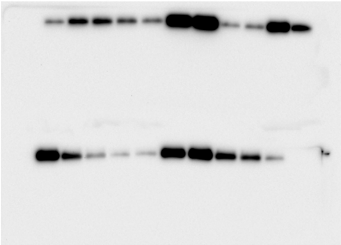

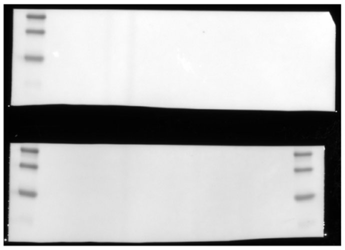

pACC Ser79

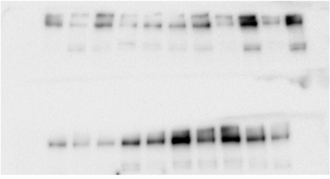

ACC

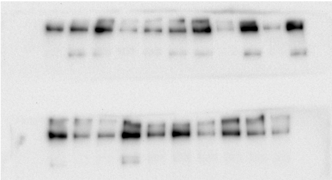

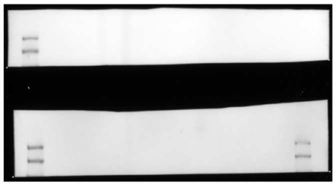

pAKT Ser473

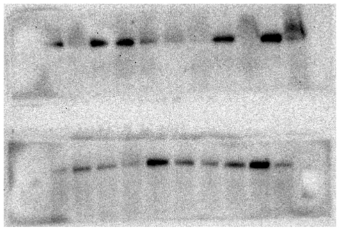

AKT

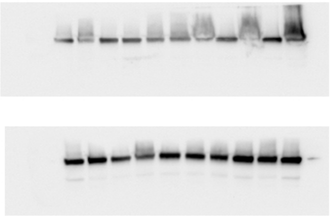

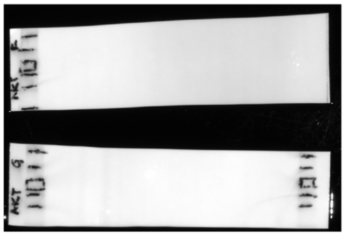

ß-Actin

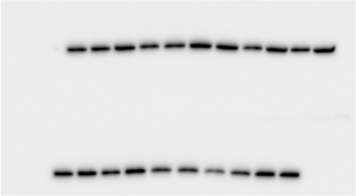

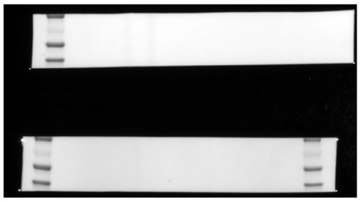

Supplement: Unedited blot and gel images [file jciinsight-10-189683-s220.pdf]
